# Transforming sickle cell haemotherapy in Africa: building safe, antigen-matched, available, and sustainable transfusion pathways

**DOI:** 10.3389/fmed.2026.1918271

**Published:** 2026-09-25

**Authors:** Emmanuel Ifeanyi Obeagu

**Affiliations:** 1 Division of Haematology, Department of Biomedical and Laboratory Science, Africa University, Mutare, Zimbabwe; 2 Department of Molecular Medicine and Haematology, School of Pathology, Faculty of Health Sciences, University of the Witwatersrand, Johannesburg, South Africa

**Keywords:** Africa, blood transfusion, healthcare sustainability, sickle cell disease, transfusion safety

## Abstract

Sickle cell disease (SCD) is a major haematological and health-system challenge in Africa, where a substantial global burden occurs alongside persistent limitations in access to safe and comprehensive transfusion services. Blood transfusion remains essential for selected acute complications, perioperative support, stroke prevention, and chronic transfusion programmes. However, effective SCD haemotherapy is constrained not only by blood shortages but also by transfusion-transmitted infections, red-cell alloimmunisation, limited antigen matching, complex immunohaematological phenotypes, iron overload, fragmented transfusion histories, and inadequate haemovigilance. This narrative review examines these interconnected challenges across the haemotherapeutic continuum and proposes practical pathways for safer, more available, and sustainable transfusion care. Evidence from published literature, transfusion guidance, SCD recommendations, and African blood-service studies was synthesised across donor recruitment, blood collection, infectious-disease screening, component preparation, compatibility testing, antigen profiling, transfusion administration, haemovigilance, and long-term monitoring. The review identifies a critical distinction between blood availability and compatible blood availability and proposes integrated solutions including sustained voluntary donor recruitment, improved inventory management, extended red-cell antigen profiling, risk-based antigen matching, selective molecular genotyping, lifelong transfusion records, antibody registries, rare-donor networks, regional reference laboratories, and strengthened haemovigilance. Rh and Kell matching should form an important foundation for frequently transfused patients, while Duffy, Kidd, MNS, and other clinically significant antigens should be incorporated according to patient risk and local capacity. Iron surveillance, appropriate chelation, patient blood management, and disease-modifying therapies can reduce cumulative transfusion-related morbidity. Africa therefore requires a transition from episodic blood provision to resilient, integrated, lifelong haemotherapeutic systems capable of improving transfusion safety, continuity, equity, and long-term outcomes for people living with SCD.

## 2 Introduction

Sickle cell disease (SCD) is one of the most important inherited haematological disorders affecting populations in Africa and represents a persistent challenge for health systems across the continent. SCD is caused predominantly by homozygosity for the *β*-globin S allele or compound heterozygosity with other pathogenic haemoglobin variants, resulting in chronic haemolysis, intermittent vaso-occlusion, anaemia, progressive organ injury, and susceptibility to life-threatening complications. Although advances in newborn screening, infection prevention, hydroxyurea, comprehensive clinical care, and emerging disease-modifying therapies have improved outcomes in some settings, substantial disparities in diagnosis, treatment access, specialist care, and supportive services remain. These disparities are particularly consequential in sub-Saharan Africa, where the largest population-level burden of SCD occurs (1, 2). Blood transfusion remains a critical component of SCD management. It can be lifesaving in severe acute anaemia, acute chest syndrome, selected neurological complications, splenic or hepatic sequestration, perioperative settings, pregnancy-associated complications, and other severe clinical circumstances. Regular transfusion programmes are also used for primary and secondary prevention of cerebrovascular complications in selected high-risk patients. Consequently, transfusion medicine is not an ancillary component of SCD care but an essential therapeutic infrastructure that may be required repeatedly across the patient's lifetime (3, 4).

The clinical value of transfusion, however, depends on the integrity of the entire haemotherapeutic pathway. This pathway begins with recruitment and retention of suitable blood donors and continues through blood collection, component preparation, infectious-disease screening, storage, inventory management, patient identification, immunohaematological testing, compatibility assessment, transfusion administration, adverse-event monitoring, and long-term follow-up. Failure at any point can compromise patient safety. Importantly, the challenge in SCD is not simply whether blood is available, but whether safe, compatible, appropriately matched, traceable, and clinically appropriate blood is available when it is needed (5, 6)**.** Blood availability remains a fundamental limitation in many African settings. Blood services frequently operate under resource constraints characterised by inadequate donor pools, seasonal variation in collection, uneven geographic distribution, limited cold-chain infrastructure, shortages of trained personnel, reagent interruptions, and constrained financing. These limitations can lead to delayed transfusion during emergencies and may disproportionately affect patients requiring chronic transfusion support. Moreover, blood availability should be distinguished from compatible-unit availability. A blood bank may possess an apparently adequate inventory while being unable to provide suitable units for an SCD patient with multiple alloantibodies or a complex red-cell antigen profile (7–9).

Transfusion safety presents another dimension of the problem. Improvements in donor selection, laboratory screening, quality management, and diagnostic technologies have substantially reduced transfusion-transmitted infection risks in many settings. Nevertheless, the quality and availability of screening systems remain heterogeneous across Africa, and continued vigilance is necessary because blood safety is dependent on the reliability of the entire testing and quality-management chain. Contemporary SCD haemotherapy therefore requires a risk-based perspective in which infectious safety is maintained while increasing attention is directed toward immunohaematological complications and long-term transfusion consequences (10). Red-cell alloimmunisation represents a particularly important challenge for chronically transfused individuals with SCD. Repeated exposure to donor erythrocyte antigens that differ from those of the recipient can induce clinically significant antibodies, increasing the difficulty of finding compatible blood and potentially precipitating delayed haemolytic transfusion reactions. The problem is further complicated by the genetic diversity of blood-group systems within African populations, including variation in the Rh system. Conventional ABO and RhD compatibility alone may therefore be insufficient for patients receiving repeated transfusions. Extended red-cell antigen profiling and, where indicated, molecular genotyping can provide a more precise foundation for transfusion planning (11).

Antigen matching must nevertheless be implemented within the realities of African blood inventories. Prophylactic matching for Rh and Kell antigens can reduce alloimmunisation in frequently transfused patients, but additional clinically significant systems—including Duffy, Kidd, and MNS—may also become important depending on the patient's antibody history, transfusion burden, antigen profile, and available donor resources. The challenge is consequently to develop risk-based and population-informed matching strategies that improve patient safety without rendering already limited blood inventories unusable (12). Repeated transfusion also creates cumulative risks beyond alloimmunisation. Transfusional iron accumulation can progressively increase body iron stores and contribute to hepatic, cardiac, and endocrine complications. Iron burden therefore requires longitudinal assessment rather than recognition only after substantial accumulation has occurred. Serum ferritin can support monitoring, while tissue-iron assessment using validated imaging approaches may provide additional information where available. Appropriate chelation, adherence support, and integration of iron monitoring into chronic transfusion programmes are consequently essential components of long-term haemotherapeutic care (13).

Haemovigilance provides the surveillance framework connecting these individual safety challenges. Systematic identification and reporting of acute and delayed transfusion reactions, alloimmunisation, transfusion-associated infections, hyperhaemolytic events, and procedural errors allow health systems to identify weaknesses and implement corrective measures. However, haemovigilance remains variably developed across African healthcare systems. Fragmented transfusion records further compound the problem because clinically important antibody histories may be lost when patients receive care at different hospitals. A lifelong transfusion record containing blood group, extended phenotype or genotype, antibody history, transfusion exposure, adverse reactions, and special blood requirements could substantially improve continuity and safety (12). Strengthening haemotherapy should not be equated with increasing transfusion exposure. Patient Blood Management and effective disease-modifying treatment can reduce avoidable transfusion requirements while preserving access to transfusion when clinically indicated. Hydroxyurea, infection prevention, newborn screening, stroke surveillance, and other components of comprehensive SCD care can reduce complications that otherwise generate transfusion demand. Emerging disease-modifying and potentially curative therapies may further change the long-term transfusion landscape, although advanced treatment pathways themselves may require sophisticated transfusion support (13). The central problem is therefore architectural rather than merely logistical. Blood availability, immunohaematological compatibility, infectious safety, haemovigilance, iron management, clinical appropriateness, digital information systems, workforce capacity, and financing are interconnected elements of one lifelong therapeutic pathway. Addressing these components separately risks producing fragmented improvements that do not translate into resilient patient care.

Against this background, this narrative review moves beyond cataloguing established transfusion complications to interrogate pathways for building resilient SCD haemotherapy in Africa. It examines the haemotherapeutic continuum from donor recruitment to lifelong patient monitoring and evaluates practical strategies for improving blood availability, transfusion safety, antigen compatibility, prevention of alloimmunisation, management of iron burden, haemovigilance, digital transfusion records, and regional transfusion capacity. Particular emphasis is placed on solutions that are clinically rational, adaptable to resource-variable African settings, and capable of integration with disease-modifying SCD care. The overarching proposition is that Africa must transition from a model centred on episodic blood provision toward safe, compatible, traceable, equitable, and sustainable lifelong haemotherapeutic systems.

### 2.1 Aim

This review aims to critically examine the current state of blood transfusion services for Sickle Cell Disease (SCD) in Africa, focusing on the key aspects of safety, availability, and sustainability.

## 3 Methods

### 3.1 Review design

This article was conducted as a narrative review designed to synthesise current evidence on haemotherapy for sickle cell disease (SCD) in Africa, with particular emphasis on the availability, safety, immunohaematological compatibility, accessibility, and sustainability of transfusion services. The review was not designed as a formal systematic review or meta-analysis because the available literature encompasses heterogeneous study designs, clinical populations, transfusion practices, health-system contexts, and outcome measures. A structured and purposive literature-search strategy was nevertheless used to maximise the identification of relevant evidence and improve transparency and reproducibility.

### 3.2 Literature search strategy

A structured search of PubMed/MEDLINE, Scopus, Web of Science, and Google Scholar was undertaken to identify relevant peer-reviewed literature and authoritative evidence concerning SCD haemotherapy. The search covered literature published from January 2000 to July 2026, with earlier seminal publications incorporated when considered important for historical or conceptual context. Additional relevant publications were identified through manual examination of the reference lists of key articles, clinical guidelines, major reviews, and authoritative transfusion medicine documents.

Search terms were developed around the major concepts of SCD, blood transfusion, immunohaematology, blood safety, and African health systems. Representative combinations included:

(“sickle cell disease” OR “sickle cell anaemia” OR “sickle cell anemia”) AND (“blood transfusion” OR haemotherapy OR hemotherapy OR “red cell transfusion” OR “chronic transfusion” OR “exchange transfusion”) AND (Africa OR “sub-Saharan Africa”)

Additional searches incorporated specific concepts including:

alloimmunisation OR alloimmunization OR “antigen matching” OR “red cell antigen” OR “blood availability” OR “blood safety” OR haemovigilance OR hemovigilance OR “transfusion reaction” OR hyperhaemolysis OR hyperhemolysis OR “iron overload” OR “blood supply” OR “red cell genotyping” OR “extended phenotyping”.

Searches were adapted to the indexing requirements of individual databases. The search strategy was deliberately broad to capture both clinical and health-system evidence relevant to the transformation of SCD haemotherapy in African settings.

## 4 Eligibility and selection of literature

Literature was considered eligible when it provided substantive information relevant to one or more of the following domains:

1. transfusion indications and clinical management of SCD;
2. acute and chronic red-cell transfusion;
3. exchange transfusion;
4. red-cell alloimmunisation;
5. antigen matching and extended phenotyping;
6. molecular blood-group genotyping;
7. delayed haemolytic transfusion reactions and hyperhaemolysis;
8. transfusion-transmitted infections and blood safety;
9. blood availability and donor recruitment;
10. blood storage, transportation, and inventory management;
11. haemovigilance and transfusion quality systems;
12. transfusion-associated iron overload;
13. patient blood management;
14. transfusion infrastructure and workforce capacity;
15. sustainability and financing of blood services; and
16. African or other resource-constrained settings with relevance to SCD haemotherapy.

Priority was given to peer-reviewed original research, clinical guidelines, consensus recommendations, authoritative reports, systematic or narrative reviews, and major transfusion medicine publications. Studies from Africa were prioritised where directly relevant. International evidence was also incorporated when African-specific evidence was limited, particularly for established principles of transfusion practice, antigen matching, alloimmunisation, exchange transfusion, and transfusion safety. Publications were excluded when they were unrelated to SCD haemotherapy, lacked substantive relevance to transfusion medicine or African blood-service challenges, or provided duplicative information without adding meaningful clinical or contextual evidence. Reference lists were manually reviewed to identify important publications not retrieved through the principal database searches.

### 4.1 Data extraction and thematic organisation

Relevant information was extracted manually by the single author from the selected literature. Extracted information included publication characteristics, study population, geographical setting, transfusion strategy, blood-safety considerations, antigen-matching approaches, alloimmunisation outcomes, blood availability, transfusion complications, infrastructure requirements, and recommendations for service improvement. Rather than applying a quantitative pooling approach, the evidence was organised into interconnected thematic domains. These themes were developed iteratively during the review process according to recurring clinical and health-system issues identified across the literature. The principal themes were:

- clinical indications for SCD haemotherapy;
- blood availability and supply dynamics;
- transfusion safety and infectious risk;
- red-cell antigen matching and immunohaematology;
- alloimmunisation and delayed haemolytic transfusion reactions;
- simple versus exchange transfusion;
- iron overload and long-term transfusion complications;
- donor recruitment and blood inventory management;
- haemovigilance and digital transfusion systems;
- infrastructure, workforce, and financing; and
- sustainable and precision haemotherapy pathways for Africa.

Evidence was compared across these domains to identify common barriers, areas of consensus, important knowledge gaps, and potential strategies for improving transfusion services.

### 4.2 Approach to evidence interpretation

The synthesis was qualitative and narrative, with emphasis on clinical relevance, consistency of evidence, applicability to African health systems, and feasibility in resource-constrained settings. Greater weight was given to established clinical guidelines, consensus recommendations, well-designed primary studies, and authoritative transfusion medicine evidence when formulating clinical or health-system recommendations. African evidence was specifically considered in relation to the continent's distinctive epidemiological, genetic, infrastructural, and health-system characteristics. Particular attention was given to the implications of African red-cell antigen diversity, limited availability of extended immunohaematological testing, chronic blood shortages, geographical disparities in transfusion access, and the need for sustainable donor and blood-service systems.

### 4.3 Quality assessment

Because this article was designed as a narrative review rather than a systematic review, a formal study-level risk-of-bias assessment or quantitative evidence grading was not undertaken. Nevertheless, the interpretation of evidence considered study design, methodological robustness, consistency of findings, clinical relevance, recency, and applicability to African settings. Where evidence was limited or heterogeneous, conclusions were presented cautiously and framed as priorities for further research rather than definitive recommendations.

### 4.4 Narrative synthesis

The final synthesis integrates clinical, laboratory, transfusion-service, and health-system evidence to develop a conceptual framework for transforming SCD haemotherapy in Africa. The framework is structured around four interdependent priorities: safe blood, antigen-matched blood, available blood, and sustainable transfusion systems. The review further considers how patient blood management, extended red-cell phenotyping and genotyping, digital transfusion records, haemovigilance, regional blood networks, specialised exchange transfusion centres, and disease-modifying SCD therapies can be integrated into a patient-centred haemotherapy model.

### 4.5 Methodological scope

As a narrative review, this study does not claim exhaustive identification of every publication on SCD transfusion in Africa. Instead, it aims to provide a clinically focused and health-system-oriented synthesis of the most relevant evidence, identify major gaps in current haemotherapy pathways, and propose practical strategies for developing safer, more compatible, more accessible, and more sustainable transfusion services across African settings.

### 4.6 Limitations

This narrative review has several limitations. First, it was conducted by a single author, which may introduce selection and interpretive bias because independent duplicate screening, data extraction, and adjudication were not performed. Second, heterogeneity in study designs, populations, transfusion practices, and healthcare resources across African countries limits direct comparison and generalizability of findings. Third, evidence on advanced approaches such as red-cell genotyping, extended antigen matching, and automated exchange transfusion remains limited in several African settings. Finally, the review synthesizes available evidence narratively rather than quantitatively; therefore, the conclusions should be interpreted as a strategic and evidence-informed framework rather than pooled estimates of effect.

#### 4.6.1 The role of blood transfusion in sickle cell disease management

Blood transfusion remains one of the most important supportive interventions in the management of SCD, particularly when acute or chronic complications exceed the patient's physiological capacity to maintain adequate oxygen delivery. The fundamental therapeutic objectives are to restore oxygen-carrying capacity, suppress the proportion of circulating sickle haemoglobin (HbS) when clinically indicated, and prevent or limit tissue injury associated with severe anaemia or vaso-occlusion. However, transfusion should not be regarded as a routine response to a low haemoglobin concentration. Its use requires careful assessment of the clinical indication, baseline haemoglobin, haemolytic status, transfusion history, alloantibody profile, and anticipated future transfusion requirements (4). In acute SCD, transfusion is primarily indicated when clinically significant anaemia develops or when complications require rapid modification of red-cell composition. Severe acute anaemia may occur during aplastic crisis, splenic or hepatic sequestration, acute haemolysis, infection, or other acute illnesses. In these circumstances, simple red-cell transfusion can rapidly increase oxygen-carrying capacity. The decision to transfuse should nevertheless consider the patient's baseline haemoglobin because individuals with SCD may tolerate haemoglobin concentrations that would appear low in individuals without chronic haemolytic anaemia. Excessive or unnecessarily rapid correction can increase blood viscosity and potentially worsen vaso-occlusive complications. The therapeutic objective should therefore be restoration of an appropriate haemoglobin concentration rather than indiscriminate normalisation (14).

Transfusion also has an important role in acute chest syndrome, one of the major causes of morbidity and mortality in SCD. Simple transfusion may be appropriate when anaemia or hypoxaemia is clinically significant, whereas red-cell exchange may be considered in severe or rapidly progressive disease, particularly when there is significant hypoxaemia, neurological involvement, or failure to respond adequately to initial management. The choice between simple transfusion and exchange transfusion depends on disease severity, haemoglobin concentration, HbS proportion, clinical trajectory, available expertise, and access to compatible red-cell units (4). Neurological complications represent another critical indication. Acute ischaemic stroke and other severe cerebrovascular events require urgent specialist management, with transfusion-based strategies designed to rapidly reduce the circulating proportion of HbS. Chronic transfusion may subsequently be used for secondary stroke prevention in appropriately selected patients. These programmes demonstrate the transition from transfusion as an emergency intervention to transfusion as a structured long-term disease-modifying strategy (15).

Perioperative transfusion represents another important application. Patients with SCD undergoing major surgery may have increased risks related to anaemia, hypoxaemia, vaso-occlusion, and acute chest syndrome. Transfusion decisions should therefore be individualised according to genotype, disease severity, baseline haemoglobin, surgical risk, previous transfusion history, and the complexity of the planned procedure. Importantly, perioperative transfusion should form part of a broader strategy incorporating hydration, oxygenation, temperature control, infection prevention, analgesia, and avoidance of other physiological triggers of sickling (16). Pregnancy presents additional complexity because maternal anaemia, placental dysfunction, vaso-occlusive complications, and obstetric emergencies can interact. Transfusion may be necessary for clinically significant anaemia or acute complications, although routine prophylactic transfusion should not be assumed to provide benefit in every pregnant patient with SCD. Individualised assessment and multidisciplinary obstetric-haematological management are essential. Chronic transfusion has a distinct role in selected patients at high risk of recurrent cerebrovascular disease and other severe complications. The objective is generally to maintain a sufficiently low HbS proportion while preserving an adequate haemoglobin concentration. Chronic transfusion can substantially reduce stroke risk in appropriately selected high-risk children, but its benefits must be balanced against cumulative complications, particularly alloimmunisation, transfusion reactions, iron overload, and increasing difficulty obtaining compatible blood (14).

The distinction between simple transfusion and red-cell exchange is therefore clinically important. Simple transfusion is more widely accessible and primarily increases red-cell mass, but repeated administration produces progressive iron accumulation. Red-cell exchange simultaneously removes a proportion of the patient's erythrocytes and replaces them with donor red cells, allowing substantial reduction of HbS while potentially limiting net iron accumulation. Its broader implementation in Africa is constrained by equipment costs, technical expertise, vascular-access requirements, blood availability, and the need for highly coordinated transfusion services. Consequently, its expansion requires health-economic assessment and infrastructure development rather than simple transplantation of high-resource models (17). A central challenge in African SCD haemotherapy is that transfusion demand exists within blood systems that may already experience shortages. Patients with SCD therefore compete for blood with individuals requiring transfusion for obstetric haemorrhage, trauma, surgery, malaria-associated anaemia, cancer, and other conditions. This makes patient blood management (PBM) particularly relevant. PBM does not mean withholding clinically necessary transfusion; rather, it seeks to prevent avoidable transfusion through optimisation of erythropoiesis, reduction of unnecessary blood loss, appropriate clinical thresholds, and use of disease-modifying therapies (16).

Hydroxyurea is particularly important in this context. By increasing fetal haemoglobin and reducing vaso-occlusive complications and other disease manifestations, hydroxyurea can reduce the frequency of clinical events that precipitate transfusion. Newborn screening, infection prevention, vaccination, penicillin prophylaxis where indicated, nutritional support, early recognition of complications, and comprehensive SCD follow-up similarly contribute indirectly to reducing transfusion dependence. Thus, strengthening disease-modifying and preventive care should be viewed as a strategy for preserving scarce blood resources while improving patient outcomes (15). The long-term transfusion trajectory must also be considered from the patient's first exposure. Every transfusion introduces the possibility of alloantibody formation, and repeated exposure can progressively narrow the pool of compatible donor units. For this reason, transfusion decisions in SCD should incorporate not only the immediate clinical indication but also the patient's future transfusion requirements. Extended red-cell antigen profiling, appropriate matching, comprehensive antibody histories, and molecular genotyping for selected patients can reduce cumulative immunohaematological risk (14). The transfusion pathway must also include systematic monitoring for adverse events. Acute haemolytic reactions, febrile reactions, allergic reactions, delayed haemolytic transfusion reactions, hyperhaemolysis, and transfusion-transmitted infections require prompt recognition and documentation. A comprehensive haemovigilance system can convert individual transfusion events into opportunities for system-wide quality improvement. This is particularly important in Africa, where patients may receive transfusions across multiple hospitals and where fragmented records can result in loss of clinically important antibody histories (Table 1) (17).

**Table 1:** The role of blood transfusion in sickle cell disease management.

Clinical indication/setting	Primary therapeutic objective	Preferred transfusion strategy	Key benefits	Major risks/limitations	Key considerations for African settings
Severe acute anaemia	Restore oxygen-carrying capacity and prevent tissue hypoxia	Simple red-cell transfusion	Rapid correction of clinically significant anaemia	Hyperviscosity if haemoglobin is increased excessively; alloimmunisation; iron accumulation	Assess against patient's steady-state haemoglobin; ensure appropriately screened and compatible blood
Aplastic crisis	Correct acute reduction in red-cell mass	Simple red-cell transfusion when clinically indicated	Rapid restoration of oxygen delivery	Transfusion reactions; alloimmunisation; iron exposure	Investigate precipitating infection and maintain accurate transfusion history
Splenic or hepatic sequestration	Restore circulating blood volume and red-cell mass	Carefully monitored simple transfusion	Corrects severe anaemia and supports haemodynamic stability	Hyperviscosity, volume overload, recurrent sequestration	Requires urgent access to compatible blood and close clinical monitoring
Acute chest syndrome	Improve oxygen delivery and reduce HbS-mediated sickling	Simple transfusion for selected moderate cases; exchange transfusion for severe/progressive disease	Improves oxygenation and reduces HbS proportion	Alloimmunisation, iron overload; exchange requires specialised resources	Early recognition, multidisciplinary management, and access to compatible units are critical
Acute neurological complications/stroke	Rapidly reduce HbS proportion and prevent further cerebral injury	Urgent red-cell exchange where feasible; simple transfusion when exchange is unavailable or inappropriate	Rapid HbS reduction and improved cerebral oxygen delivery	Alloimmunisation, transfusion reactions, technical and blood-supply requirements	Requires rapid referral, specialist transfusion capacity, and reliable access to appropriately matched blood
Primary stroke prevention in high-risk patients	Maintain low HbS exposure and reduce recurrent cerebrovascular injury	Regular chronic transfusion programme	Substantially reduces stroke risk in appropriately selected high-risk patients	Iron overload, alloimmunisation, transfusion dependence	Requires sustained blood supply, systematic monitoring, and long-term financing
Secondary stroke prevention	Prevent recurrent cerebrovascular events	Regular chronic transfusion, often with exchange where available	Reduces risk of recurrent stroke	Cumulative iron burden, alloimmunisation and lifelong transfusion dependence	Requires reliable long-term follow-up and comprehensive transfusion records
Perioperative management	Reduce risk of severe anaemia, hypoxaemia, vaso-occlusion and acute chest syndrome	Individualised simple transfusion or exchange transfusion according to surgical risk	Improves perioperative physiological reserve in selected patients	Transfusion reactions, alloimmunisation, iron exposure	Requires preoperative planning and availability of antigen-compatible blood
Pregnancy-associated severe anaemia or acute complications	Support maternal oxygen delivery and treat clinically significant complications	Individualised red-cell transfusion	Corrects clinically important anaemia and supports maternal/fetal stability	Alloimmunisation, transfusion reactions, iron accumulation	Requires coordinated obstetric, haematological and transfusion care
Recurrent severe SCD complications	Reduce disease-related morbidity when conventional management is inadequate	Individualised chronic transfusion or exchange programme	May reduce frequency of selected severe complications	Long-term transfusion burden and iron overload	Should be integrated with hydroxyurea and other disease-modifying therapies
Red-cell exchange programmes	Reduce HbS proportion while minimising net iron accumulation	Automated or manual red-cell exchange	Effective HbS reduction with less net iron loading than repeated simple transfusion	High resource requirement; donor-unit demand; vascular-access challenges	Expansion requires equipment, trained personnel, reliable blood supply and cost-effectiveness assessment
Chronic transfusion-dependent SCD	Maintain therapeutic HbS suppression and adequate haemoglobin	Scheduled simple transfusion or exchange transfusion	Long-term prevention of selected complications	Alloimmunisation, iron overload, delayed haemolytic reactions	Requires lifelong transfusion records, antibody surveillance, iron monitoring and sustainable financing
Patient Blood Management	Minimise avoidable transfusion while preserving access when clinically necessary	Optimisation of erythropoiesis, prevention of blood loss and evidence-based transfusion	Conserves blood resources and reduces cumulative transfusion exposure	Requires clinical coordination and monitoring	Particularly important where blood supplies are constrained
Integration with disease-modifying therapy	Reduce complications and transfusion dependence	Hydroxyurea and other disease-modifying therapies alongside indicated transfusion	Can reduce vaso-occlusive events and transfusion demand	Access, adherence and monitoring challenges	Strengthening disease-modifying treatment can indirectly improve blood-system sustainability
Long-term transfusion surveillance	Prevent and detect cumulative transfusion complications	Antibody screening, extended antigen profiling, haemovigilance and iron monitoring	Improves safety and continuity of care	Requires laboratory capacity and longitudinal records	Electronic transfusion histories, regional reference laboratories and rare-donor registries can strengthen the pathway

HbS, sickle haemoglobin; SCD, sickle cell disease; PBM, patient blood management.

Iron management is equally important in patients receiving repeated transfusions. Each unit of transfused red cells contributes additional iron, and cumulative exposure can produce progressive iron accumulation. Chronic transfusion programmes should therefore incorporate regular assessment of transfusion burden and iron status, appropriate use of chelation therapy, monitoring of treatment adherence, and assessment of organ-specific iron toxicity where resources permit. Iron management should be viewed as an integral component of transfusion care rather than a complication addressed only after severe iron overload has developed.

The role of transfusion in SCD is therefore best conceptualised as a continuum rather than an isolated procedure:

clinical indication → transfusion risk assessment → compatible blood selection → appropriate administration → acute monitoring → haemovigilance → antibody surveillance → iron monitoring → reassessment of future transfusion need.

This approach has particular relevance for Africa because the effectiveness of transfusion depends on the capacity of the surrounding blood system. A clinically appropriate transfusion strategy cannot succeed if compatible blood is unavailable, transfusion histories are inaccessible, laboratory testing is unreliable, or post-transfusion complications cannot be monitored.

### 4.7 Clinical indications and therapeutic role of hemotherapy in SCD

Haemotherapy occupies a critical but highly selective position in the management of SCD. Its therapeutic value extends beyond correction of anaemia to include restoration of oxygen-carrying capacity, reduction of circulating sickle haemoglobin (HbS), prevention of cerebrovascular complications, and support during selected acute, surgical, and obstetric emergencies. However, transfusion is not a substitute for comprehensive disease-modifying treatment and should not be triggered solely by a numerical haemoglobin threshold. The decision to transfuse should integrate clinical severity, change from the patient's steady-state haemoglobin, HbS proportion, evidence of organ dysfunction, transfusion history, alloantibody status, and availability of appropriately compatible blood (18). Acute severe anaemia is one of the clearest indications for red-cell transfusion in SCD. Patients may develop abrupt reductions in haemoglobin because of aplastic crisis, acute sequestration, accelerated haemolysis, infection, or other acute illnesses. In these circumstances, simple red-cell transfusion can rapidly restore oxygen-carrying capacity and prevent tissue hypoxia. The therapeutic objective should be restoration toward the patient's clinically appropriate baseline rather than indiscriminate normalisation of haemoglobin. Excessive post-transfusion haemoglobin concentrations can increase blood viscosity, particularly in patients with relatively high baseline haemoglobin concentrations. Careful assessment of volume status and haemodynamic condition is therefore essential (19).

Acute chest syndrome (ACS) is a major cause of morbidity and mortality in SCD and represents an important indication for haemotherapy in selected patients. Transfusion may improve oxygen delivery and reduce the proportion of circulating HbS-containing erythrocytes. Simple transfusion can be appropriate for patients with clinically significant anaemia or moderate disease, whereas red-cell exchange should be considered in severe or rapidly progressive ACS, particularly when there is significant hypoxaemia, neurological involvement, or inadequate response to initial treatment. The decision should be based on disease severity and trajectory rather than a single laboratory value. In African settings, early recognition is particularly important because delayed referral and limited access to exchange transfusion may narrow therapeutic options. Strengthening regional referral pathways and ensuring access to appropriately matched blood are therefore integral to ACS management (20, 21). Acute ischaemic stroke and other severe neurological complications require urgent intervention because irreversible neurological injury can occur rapidly. Transfusion-based therapy aims to reduce the circulating HbS proportion and improve tissue oxygen delivery. Red-cell exchange is generally preferred where technically feasible because it can rapidly lower HbS without producing excessive increases in blood viscosity or red-cell mass. Where exchange transfusion is unavailable, appropriately administered simple transfusion may provide an alternative depending on the clinical circumstances. The effectiveness of this pathway depends not only on clinical expertise but also on rapid access to compatible blood, reliable immunohaematology testing, and efficient communication between emergency, haematology, neurology, and transfusion teams (22, 23).

Transfusion has an established role in selected patients at increased risk of cerebrovascular complications. Children identified through appropriate cerebrovascular screening as being at high risk may benefit from regular transfusion programmes, while patients with previous stroke may require chronic transfusion for secondary prevention. The therapeutic objective is to maintain a sufficiently low proportion of HbS and adequate haemoglobin over time. This transforms transfusion from an emergency intervention into a structured preventive therapy. However, chronic transfusion creates cumulative risks, particularly alloimmunisation and iron overload. Consequently, long-term programmes should incorporate extended red-cell antigen profiling, appropriate antigen matching, antibody surveillance, iron monitoring, and consideration of disease-modifying alternatives where clinically appropriate (24). Acute splenic or hepatic sequestration can cause rapid reductions in circulating red-cell mass and, in severe cases, haemodynamic instability. Red-cell transfusion may be lifesaving when anaemia is clinically significant. Because sequestration can alter circulating blood volume and haemoglobin rapidly, transfusion should be carefully monitored to avoid excessive haemoglobin correction and hyperviscosity. Recurrent episodes should prompt consideration of preventive strategies and comprehensive SCD management rather than repeated emergency transfusion alone (25). Aplastic crises, often associated with transient suppression of erythropoiesis, can produce profound anaemia in individuals who already have shortened red-cell survival. Transfusion may be required when the reduction in haemoglobin is clinically significant or associated with symptoms or physiological compromise. Management should simultaneously address the underlying precipitating condition and avoid unnecessary transfusion once endogenous erythropoiesis recovers (26).

Surgical procedures can increase the risk of vaso-occlusion, acute chest syndrome, hypoxaemia, and other SCD-related complications. Preoperative transfusion may therefore be considered for selected patients undergoing procedures associated with substantial physiological stress or blood loss. The decision should be individualised according to genotype, baseline haemoglobin, disease severity, previous complications, transfusion history, and surgical risk. Haemotherapy should form part of a broader perioperative strategy encompassing adequate oxygenation, temperature control, hydration, analgesia, infection prevention, and minimisation of surgical blood loss. Perioperative transfusion should not be applied uniformly to every patient with SCD. Avoiding unnecessary exposure is particularly important in resource-constrained settings because each transfusion consumes a scarce blood resource and contributes to cumulative immunohaematological risk (27). Pregnancy in SCD requires coordinated obstetric and haematological care. Transfusion may be indicated for severe symptomatic anaemia, major haemorrhage, acute chest syndrome, stroke, or other serious complications. Routine prophylactic transfusion should not be assumed to benefit every pregnant patient. When transfusion is required, maternal antibody history and red-cell antigen compatibility are particularly important because alloimmunisation can have implications for both transfusion management and pregnancy outcomes. Early antenatal identification of transfusion requirements therefore provides an opportunity for proactive blood planning (28).

Chronic transfusion is appropriate for selected patients with recurrent severe complications, particularly those requiring long-term cerebrovascular protection. Its principal therapeutic mechanism is suppression of the proportion of circulating HbS-containing erythrocytes while maintaining adequate oxygen-carrying capacity. Although effective in appropriately selected patients, chronic transfusion creates a cumulative therapeutic burden. Each additional transfusion increases exposure to donor antigens and adds to total iron accumulation. Consequently, chronic transfusion should be viewed as a long-term treatment programme, requiring regular review of clinical benefit, transfusion intensity, antibody status, iron burden, and availability of alternative disease-modifying approaches (29). The choice between simple transfusion and red-cell exchange depends on the clinical objective. Simple transfusion primarily increases red-cell mass and is generally more accessible. It is particularly useful when correction of clinically significant anaemia is the primary objective. Its major limitation during repeated use is cumulative iron loading. Red-cell exchange directly replaces a proportion of the patient's erythrocytes with donor erythrocytes and can achieve rapid HbS reduction while limiting net iron accumulation. It is particularly valuable in selected severe acute complications and chronic programmes where substantial HbS suppression is required. However, exchange transfusion requires specialised equipment, trained personnel, appropriate vascular access, reliable donor-unit availability, and sophisticated transfusion coordination. In Africa, its implementation should therefore be accompanied by investment in infrastructure, workforce development, blood inventory systems, and regional referral networks (27).

The therapeutic decision to transfuse should include consideration of the patient's future transfusion requirements. SCD patients may receive blood repeatedly over many years, making alloimmunisation an important cumulative hazard. Patients requiring recurrent transfusion should therefore have their red-cell antigen profile documented whenever feasible. Risk-based matching should incorporate Rh and Kell antigens as an important foundation, while clinically significant antigens in systems such as Duffy, Kidd, and MNS should be considered according to antibody history, patient risk, and local capacity. For patients with multiple antibodies, rare phenotypes, or complex Rh variants, molecular genotyping can provide additional information and facilitate more accurate donor selection (Table 2) (28, 29). The long-term benefits of transfusion must be balanced against transfusional iron accumulation. Patients receiving regular transfusion require systematic assessment of iron burden and appropriate intervention when clinically indicated. A comprehensive haemotherapeutic programme should therefore link:

**Table 2:** Clinical indications and therapeutic role of haemotherapy in sickle cell disease.

Clinical indication	Pathophysiological problem	Therapeutic objective	Preferred haemotherapeutic approach	Key clinical considerations	Major long-term implications
Severe acute anaemia	Acute fall in red-cell mass from haemolysis, infection, aplasia, sequestration, or other causes	Restore oxygen-carrying capacity and prevent tissue hypoxia	Simple red-cell transfusion when clinically indicated	Consider baseline/steady-state Hb, symptoms, haemodynamic status, and rate of Hb decline; avoid excessive Hb correction	Alloimmunisation, iron accumulation, transfusion reactions
Aplastic crisis	Transient suppression of erythropoiesis, commonly during acute infection	Temporarily restore circulating red-cell mass until marrow recovery	Simple red-cell transfusion for clinically significant anaemia	Identify and treat underlying cause; reassess transfusion need as erythropoiesis recovers	Usually limited cumulative risk if transfusion exposure is appropriately controlled
Splenic/hepatic sequestration	Acute pooling of erythrocytes in spleen or liver causing severe anaemia and possible hypovolaemia	Restore circulating red-cell mass and haemodynamic stability	Carefully monitored simple transfusion	Avoid excessive correction because sequestered cells may return to circulation; monitor Hb and volume status closely	Recurrent episodes may increase cumulative transfusion exposure
Acute chest syndrome	Pulmonary vaso-occlusion, inflammation, hypoxaemia and impaired oxygen delivery	Improve oxygenation and reduce circulating HbS burden	Simple transfusion for selected moderate disease; red-cell exchange for severe/progressive disease	Early recognition is essential; assess hypoxaemia, anaemia, clinical trajectory, and response to initial therapy	Alloimmunisation, iron overload, recurrent transfusion dependence
Acute ischaemic stroke	Cerebral vaso-occlusion and impaired tissue oxygenation	Rapidly reduce HbS proportion and improve cerebral oxygen delivery	Urgent red-cell exchange preferred where available; simple transfusion when exchange is unavailable or unsuitable	Requires emergency multidisciplinary management and rapid access to compatible blood	Often necessitates long-term secondary stroke-prevention transfusion
Primary stroke prevention	High risk of cerebrovascular injury identified through appropriate screening	Maintain low HbS exposure and prevent first stroke	Regular chronic transfusion programme in appropriately selected high-risk patients	Requires structured monitoring and adherence to scheduled transfusions	Iron overload, alloimmunisation, long-term blood demand
Secondary stroke prevention	Previous stroke with high risk of recurrence	Prevent recurrent cerebrovascular events	Chronic transfusion or red-cell exchange according to patient and resource context	Long-term HbS suppression, antibody monitoring, and iron surveillance are essential	Lifelong transfusion burden unless an appropriate alternative strategy becomes available
Perioperative management	Increased risk of anaemia, hypoxaemia, vaso-occlusion and acute chest syndrome during surgery	Reduce perioperative morbidity in selected high-risk procedures	Individualised simple transfusion or exchange transfusion	Consider genotype, baseline Hb, surgical risk, previous complications, and transfusion history	Additional alloimmunisation and iron exposure
Pregnancy with severe anaemia or acute complication	Maternal anaemia or acute SCD/obstetric complication compromising maternal–fetal oxygen delivery	Stabilise maternal physiology and treat clinically significant anaemia	Individualised red-cell transfusion	Requires coordinated obstetric, haematological and transfusion management; routine prophylactic transfusion is not universally indicated	Alloimmunisation may complicate future transfusion and pregnancy management
Recurrent severe SCD complications	Persistent disease activity despite standard supportive care	Reduce frequency or severity of selected complications	Individualised chronic transfusion or exchange programme	Reassess disease-modifying treatment, adherence, and indication periodically	Cumulative iron burden and alloimmunisation
Chronic transfusion programme	Sustained risk of severe complications requiring HbS suppression	Maintain therapeutic HbS reduction and adequate oxygen-carrying capacity	Scheduled simple transfusion or red-cell exchange	Requires extended antigen profiling, antibody surveillance, iron monitoring, and documented transfusion targets	Progressive transfusion dependence, iron overload, alloimmunisation
Red-cell exchange therapy	Need for rapid HbS reduction while limiting net iron accumulation	Replace HbS-containing erythrocytes while maintaining appropriate Hb	Automated or manual red-cell exchange	Requires specialised equipment, trained staff, vascular access, compatible blood, and technical expertise	Lower net iron accumulation than repeated simple transfusion but substantial resource requirements
Patient Blood Management	Avoidable transfusion exposure and inefficient use of limited blood resources	Optimise erythropoiesis, minimise blood loss, and ensure appropriate transfusion	Evidence-based, individualised transfusion strategy	Transfusion should be based on clinical indication rather than Hb value alone	Reduced cumulative transfusion exposure and improved blood-resource stewardship
Disease-modifying therapy integration	Recurrent SCD complications generating preventable transfusion requirements	Reduce disease activity and transfusion dependence	Hydroxyurea and other disease-modifying therapies alongside indicated haemotherapy	Requires access, adherence, laboratory monitoring, and long-term follow-up	Potential reduction in transfusion demand and blood-system pressure
Alloimmunisation prevention	Exposure to non-self red-cell antigens during repeated transfusion	Preserve future compatibility and prevent haemolytic reactions	Extended antigen profiling and risk-based antigen matching	Rh and Kell matching as an important foundation; consider Duffy, Kidd, MNS and other clinically significant antigens according to risk and capacity	Reduced antibody formation and improved future access to compatible blood
Complex immunohaematology	Multiple alloantibodies, rare phenotypes, or complex Rh variants	Identify compatible donor units and prevent haemolytic reactions	Molecular red-cell genotyping and reference immunohaematology support	Particularly valuable after recent transfusion or when serological phenotyping is unreliable	Facilitates long-term compatible blood provision
Transfusional iron overload	Progressive iron accumulation from repeated red-cell exposure	Prevent organ damage while maintaining necessary transfusion therapy	Serial iron assessment, appropriate chelation, and consideration of exchange transfusion	Ferritin trends can support monitoring; tissue-iron assessment should be used where available and clinically appropriate	Hepatic, cardiac and endocrine morbidity if inadequately managed
Haemovigilance and long-term surveillance	Acute/delayed reactions, alloimmunisation, hyperhaemolysis and transfusion errors	Detect complications early and improve transfusion-system safety	Structured haemovigilance and lifelong transfusion documentation	Record antibody history, transfusion exposure, reactions, phenotype/genotype and special requirements	Improved continuity of care and reduced recurrent transfusion-related harm

Hb, haemoglobin; HbS, sickle haemoglobin; SCD, sickle cell disease.

transfusion exposure → iron-risk assessment → serial monitoring → chelation where indicated → adherence assessment → evaluation of organ iron burden where feasible.

This approach prevents iron overload from becoming an unrecognised cumulative consequence of otherwise successful transfusion therapy. Patient Blood Management provides an important framework for ensuring that transfusion is used appropriately. In SCD, PBM involves optimising endogenous erythropoiesis, treating reversible contributors to anaemia, preventing avoidable blood loss, using evidence-based transfusion indications, and integrating disease-modifying therapies. The objective is not simply to reduce the number of transfusions. Rather, it is to ensure that the right patient receives the right blood product at the right time, with the lowest reasonable cumulative transfusion risk**.** This principle is particularly relevant in Africa, where blood supplies are frequently constrained and unnecessary transfusion may reduce availability for patients with urgent clinical needs (27). Haemotherapy should operate within a broader SCD treatment strategy. Hydroxyurea and other disease-modifying therapies can reduce vaso-occlusive complications and other clinical events that may otherwise lead to hospitalisation and transfusion. Newer therapeutic approaches, including agents targeting haemoglobin polymerisation and other pathways, as well as potentially curative cellular and gene-based interventions, may progressively alter transfusion requirements. Nevertheless, these approaches do not eliminate the need for robust transfusion services because patients may continue to require blood support during severe complications or advanced therapeutic interventions (28).

The clinical role of haemotherapy in SCD should ultimately be understood as a continuum:

clinical assessment → indication for transfusion → immunohaematological risk assessment → selection of compatible blood → safe administration → acute monitoring → haemovigilance → antibody surveillance → iron monitoring → reassessment of future transfusion requirements.

This approach moves SCD transfusion care beyond the traditional concept of simply providing blood during an acute episode. In Africa, where blood resources are limited and specialist transfusion capacity varies considerably, this integrated model provides an opportunity to improve both patient safety and health-system efficiency. The central therapeutic principle is therefore not maximal transfusion, but strategically appropriate transfusion**.** Haemotherapy should be delivered when the expected clinical benefit outweighs cumulative risks, using the safest and most appropriately matched blood available, while simultaneously strengthening disease-modifying treatment and preventive care to reduce avoidable transfusion dependence (29).

Blood transfusion is a lifesaving component of SCD management, but its therapeutic effectiveness depends fundamentally on the safety and reliability of the blood-service infrastructure supporting it. In Africa, transfusion safety must be understood within a heterogeneous health-system environment characterised by differences in blood-service organisation, donor availability, laboratory capacity, regulatory oversight, financing, workforce, technology, and geographical access. The relevant safety question is therefore broader than whether donated blood has been tested. A safe transfusion system must ensure that blood is appropriately collected, correctly identified, adequately screened, correctly processed and stored, immunohaematologically compatible, traceable, appropriately administered, and continuously monitored for adverse outcomes (18). For people with SCD, these requirements are particularly important because transfusion may occur repeatedly over many years. A safety failure that appears clinically minor during an isolated transfusion can become consequential when repeated exposure produces alloimmunisation, delayed haemolysis, iron accumulation, or progressively limited access to compatible blood. Consequently, African blood-transfusion services require a life-course perspective in which the immediate safety of each transfusion is considered alongside its cumulative consequences (19).

African blood-transfusion systems vary considerably in their organisational structure and technical capacity. Some countries have relatively centralised national blood services with established quality-management systems, while others depend on decentralised hospital blood banks operating with variable levels of technical and regulatory support. Rural and peripheral facilities may face additional challenges involving refrigeration, electricity supply, transportation, reagent availability, equipment maintenance, and access to appropriately trained laboratory personnel (20). This heterogeneity has direct implications for SCD care. Patients frequently require transfusion at secondary or tertiary hospitals, but complex immunohaematological investigations may only be available at specialised centres. Delays in obtaining antibody identification, antigen-negative units, or expert consultation can therefore become clinically important during emergencies (21). A resilient system requires a tiered blood-service architecture in which basic transfusion services are available locally while complex testing, reference immunohaematology, molecular genotyping, and rare-donor support are concentrated in appropriately equipped regional or national centres. Such a hub-and-spoke model can improve access without requiring every hospital to maintain expensive specialised technologies (22).

Blood availability remains one of the most important determinants of transfusion safety because a clinically indicated transfusion that cannot be delivered promptly may result in preventable morbidity. Blood shortages may arise from inadequate donor recruitment, seasonal fluctuations, limited collection capacity, high clinical demand, transportation problems, or inappropriate inventory management (23). For SCD, the concept of availability should extend beyond the number of units physically present in a blood bank. The clinically relevant question is whether an appropriate unit is available for the individual patient. An SCD patient with multiple alloantibodies may require antigen-negative blood that is considerably more difficult to identify than a routinely compatible ABO/RhD unit (24). Therefore, blood-system performance should increasingly be assessed in terms of compatible blood availability rather than total blood inventory alone. This requires accurate patient antigen profiles, reliable donor antigen databases, efficient communication between hospitals and blood centres, and mechanisms for locating rare compatible units.

A stable supply of safe blood depends on a reliable donor population. Voluntary non-remunerated donation remains a fundamental component of sustainable blood services. Recruitment strategies should prioritise repeat donation and donor retention rather than relying primarily on emergency replacement donation (25). Community engagement through schools, universities, workplaces, professional organisations, faith-based groups, and civil-society networks can strengthen donor recruitment. However, recruitment must be accompanied by appropriate donor education, informed consent, confidential health assessment, and protection of donor wellbeing (26). Repeat donors may also contribute to improved blood safety because established donor histories can facilitate consistent eligibility assessment and monitoring. Nevertheless, all donors require appropriate screening at every donation according to national policies and applicable standards.

Transfusion-transmitted infections (TTIs) remain an important component of blood safety, although the magnitude of risk has declined substantially in many settings following improvements in donor selection, laboratory screening, quality assurance, and implementation of increasingly sensitive diagnostic methods. Relevant pathogens include HIV, hepatitis B virus, hepatitis C virus, syphilis, and other infections according to local epidemiology and national policy (20). The residual risk of infection depends on multiple components of the transfusion pathway. Effective safety requires appropriate donor selection, accurate specimen identification, validated assays, reliable reagent supply, equipment maintenance, trained personnel, internal quality control, external quality assessment, appropriate handling of reactive donations, and traceability (21). The continued presence of TTI risk in some African settings should therefore not be interpreted simply as a problem of inadequate testing technology. It may also reflect weaknesses in the surrounding quality-management system. A highly sensitive assay cannot compensate for poor sample identification, reagent interruptions, equipment failure, inadequate staff competency, or failure to remove reactive units from the transfusion inventory.

Serological screening remains central to blood safety because it can be implemented across a broad range of healthcare environments. Nucleic acid testing may further reduce residual infectious risk for selected pathogens by shortening the diagnostic window period, but its implementation requires substantial investment in equipment, reagents, infrastructure, staff training, maintenance, and quality assurance. African blood services should therefore adopt risk-based and context-appropriate diagnostic strategies rather than assuming that the most technologically advanced platform is automatically the most sustainable. Where molecular screening is introduced, it should complement rather than replace the broader quality-management architecture (27). Patient and specimen identification represents one of the most preventable sources of transfusion-related harm. Incorrect patient identification, mislabelled samples, transcription errors, and inappropriate blood-unit selection can result in incompatible transfusion even when the laboratory testing itself is technically accurate. Robust identification procedures should therefore be incorporated at every stage, from sample collection to bedside administration. Standardised patient identification, barcode systems where feasible, independent verification, clearly documented transfusion requests, and traceable blood-unit labelling can reduce preventable errors. These safeguards are particularly important in high-volume hospitals where emergency transfusion decisions are frequently made under considerable time pressure (28).

ABO and RhD compatibility forms the basic foundation of safe red-cell transfusion, but it may be insufficient for individuals with SCD who require repeated exposure to donor erythrocytes. Differences between recipient and donor red-cell antigens can lead to alloantibody formation and subsequent difficulty obtaining compatible blood. Extended red-cell antigen profiling should therefore be incorporated into the management of patients likely to receive repeated transfusions. This should include clinically relevant systems such as Rh, Kell, Duffy, Kidd, and MNS, alongside other antigens identified through antibody screening and transfusion history. The African context is particularly important because blood-group antigen frequencies and genetic variants may differ substantially from those represented in donor populations used to develop conventional transfusion strategies elsewhere. Complex Rh variants are an important example. Molecular genotyping can provide valuable information when recent transfusion, weak antigen expression, serological discrepancies, or variant alleles make conventional phenotyping unreliable (29). Alloimmunisation is one of the major long-term safety concerns for repeatedly transfused patients with SCD. Exposure to donor antigens absent from the recipient can result in the development of clinically significant antibodies. Once formed, these antibodies may complicate future transfusion by reducing the number of compatible units available. The problem is compounded by the potential disappearance of antibody titres below routine detection thresholds. A patient may therefore have a clinically important historical antibody even when the antibody is not detectable during a subsequent pre-transfusion test. This creates a strong argument for permanent antibody documentation. Delayed haemolytic transfusion reactions can occur when previously formed antibodies reappear following re-exposure. In SCD, such reactions can be difficult to distinguish from worsening haemolysis associated with the underlying disease. A comprehensive transfusion history, access to previous antibody results, and timely post-transfusion investigation are therefore essential (30).

Hyperhaemolysis represents a severe transfusion-associated complication in which destruction may involve both donor and autologous erythrocytes. Recognition can be challenging because the clinical picture may resemble progression of the underlying SCD. The potential severity of this complication reinforces the importance of careful transfusion indication, detailed antibody history, appropriate laboratory investigation, and specialist consultation when haemolysis occurs unexpectedly after transfusion. African transfusion services should therefore develop referral mechanisms through which complex haemolytic reactions can be investigated by regional or national reference laboratories (31). Maintaining appropriate storage conditions is essential for preserving blood-component quality. Interruptions in refrigeration, unreliable electricity, inadequate temperature monitoring, equipment failure, and transportation delays can compromise blood products. This challenge is particularly relevant in geographically dispersed healthcare systems. Strengthening blood-service resilience requires validated storage equipment, temperature monitoring, backup power systems, preventive maintenance, contingency plans, and reliable transportation. Component separation can also improve blood utilisation by allowing one donation to provide different therapeutic products for appropriate patients. However, component preparation requires suitable equipment, trained staff, quality assurance, and appropriate storage capacity (26).

Haemovigilance is essential for identifying adverse transfusion events and converting individual clinical incidents into system-wide learning. A functional haemovigilance programme should capture acute and delayed transfusion reactions, alloimmunisation, haemolysis, transfusion-transmitted infections, procedural errors, and other significant adverse events. The value of haemovigilance extends beyond reporting. Each event should trigger appropriate investigation, root-cause analysis where indicated, corrective and preventive actions, and feedback to clinical and laboratory teams. In many resource-constrained settings, under-reporting may result from limited awareness, inadequate reporting systems, fear of blame, or insufficient staffing. Developing a non-punitive safety culture is therefore essential. One of the most important but potentially addressable safety problems in SCD is fragmentation of transfusion information. Patients may receive blood at multiple hospitals, particularly when they travel, relocate, or seek emergency care. If previous transfusions and antibodies are not accessible, a clinically important antibody may be missed. A lifelong transfusion record should ideally contain ABO/RhD status, extended phenotype or genotype, antibody history, transfusion dates, transfusion reactions, special compatibility requirements, and relevant laboratory findings. Electronic transfusion databases could substantially improve this process. Where electronic systems are not yet feasible, a standardised patient-held transfusion record can provide an interim solution (32).

Conventional inventory management focuses primarily on maintaining adequate numbers of blood units and reducing wastage. SCD requires a more sophisticated approach because some patients need specific antigen-negative units. National or regional donor databases containing extended antigen information can help identify compatible units. Rare-donor registries can become particularly valuable for patients with multiple alloantibodies or uncommon antigen profiles. Regional collaboration is important because a rare compatible phenotype may not be represented sufficiently within one hospital or even one country. Establishing mechanisms for secure sharing of compatible blood between institutions can therefore transform individual blood shortages into manageable network-level problems (18). Blood safety ultimately depends on human expertise. Medical laboratory scientists, transfusion specialists, clinicians, nurses, blood-bank technologists, quality managers, biomedical engineers, and other personnel require appropriate training and continuing professional development. Competency should encompass technical laboratory procedures as well as immunohaematology, transfusion reaction investigation, haemovigilance, quality management, inventory control, and SCD-specific transfusion principles. Retention of skilled personnel is equally important. High staff turnover can undermine institutional knowledge and compromise quality systems (20).

A comprehensive quality-management system should encompass standard operating procedures, equipment validation and maintenance, reagent management, internal quality control, external quality assessment, staff competency assessment, documentation, non-conformance management, and corrective and preventive action. Regulatory oversight should ensure that blood establishments meet appropriate standards and that clinical transfusion practices are subject to governance through hospital transfusion committees or equivalent structures. Importantly, quality management should be viewed as an ongoing process rather than a one-time accreditation exercise (21). Blood safety also involves ensuring that transfusion is clinically appropriate. Unnecessary transfusion exposes patients to avoidable risks and consumes limited blood resources. Patient Blood Management provides a framework for optimising endogenous erythropoiesis, reducing preventable blood loss, and ensuring evidence-based transfusion decisions. In SCD, this should be integrated with hydroxyurea and other disease-modifying therapies that can reduce the frequency of complications requiring transfusion. The goal is not simply to reduce transfusion rates. Rather, it is to ensure that scarce blood resources are directed toward patients for whom transfusion provides meaningful clinical benefit. For patients receiving repeated transfusion, iron accumulation represents a cumulative safety problem. Unlike infection or acute incompatibility, iron overload may remain clinically silent for years before producing organ dysfunction. Transfusion programmes should therefore incorporate longitudinal iron monitoring and appropriate chelation. Where resources permit, more direct assessment of tissue iron can improve risk stratification. Iron management should be integrated into the initial design of chronic transfusion programmes rather than introduced only after substantial iron accumulation has occurred (22).

Digital technologies offer opportunities to strengthen multiple components of transfusion safety. Electronic blood-bank systems can improve traceability, inventory management, antibody documentation, and compatibility workflows. Interoperable health records could ensure that a patient's transfusion history follows them across institutions. Data-driven forecasting may also improve blood collection and inventory allocation by predicting periods of increased demand and identifying patterns of blood wastage. Molecular red-cell genotyping represents another important technological opportunity, particularly for patients with complex Rh variants or multiple alloantibodies. However, technological expansion should be guided by clinical need and sustainability. Digital systems and molecular diagnostics should strengthen—not replace—basic blood-service infrastructure (23). The safety challenges confronting African blood-transfusion services are interconnected. Blood shortages increase pressure on clinicians to use whatever compatible units are immediately available. Limited antigen typing increases alloimmunisation risk. Alloimmunisation subsequently reduces the pool of compatible units, making future shortages more clinically consequential. Fragmented records can compound the problem by obscuring previous antibody histories. Repeated transfusion then increases iron burden, while weak haemovigilance may prevent systematic recognition of these complications. The appropriate response is therefore an integrated safety architecture rather than isolated interventions. A resilient model should connect:

safe donor recruitment → reliable blood collection → rigorous infectious screening → quality-controlled processing and storage → extended patient antigen profiling → risk-based antigen matching → safe administration → haemovigilance → lifelong antibody documentation → iron monitoring → inventory and rare-donor management.

This architecture should be supported by sustainable financing, trained personnel, appropriate regulation, regional reference laboratories, and integration with comprehensive SCD care.

For SCD, the ultimate measure of blood-service safety is not simply the number of units collected or the proportion of donations screened. It is whether a patient can obtain safe, compatible, clinically appropriate blood when needed without accumulating preventable long-term harm. The African context demands solutions that are both scientifically sound and operationally realistic. Universal implementation of highly sophisticated technologies may not be immediately feasible, but meaningful improvements can be achieved through phased strategies. These include strengthening voluntary donation, ensuring reliable infectious screening, establishing extended antigen profiling for frequently transfused patients, maintaining permanent antibody histories, developing regional immunohaematology capacity, improving haemovigilance, integrating iron monitoring, and creating stronger communication between clinical services and blood establishments (26). The transition should therefore be from a reactive transfusion service to a proactive haemotherapeutic system. Such a system recognises that transfusion safety begins before blood is collected and continues throughout the patient's lifetime. For people living with SCD, this life-course approach provides the foundation for reducing avoidable transfusion complications while preserving access to haemotherapy when it is most clinically necessary (Table 3) (32).

**Table 3:** Safety concerns in African blood transfusion services and strategic pathways for sickle cell disease haemotherapy.

Priority domain	Major safety concern	Strategic pathway for SCD haemotherapy
Blood availability	Unpredictable supply and regional shortages	Strengthen voluntary donation, donor retention, inventory management, and regional blood-sharing networks
Transfusion-transmitted infections	Risk of HIV, HBV, HCV and other infections	Strengthen donor selection, quality-assured screening, component processing, and haemovigilance
ABO/Rh compatibility	Inadequate compatibility testing in resource-constrained settings	Ensure reliable ABO/RhD typing, antibody screening, and crossmatching before transfusion
Alloimmunization	Repeated transfusion increases antibody formation	Implement preventive Rh and Kell matching, with extended matching for high-risk patients
Complex Rh genetics	RH variants may complicate serological matching in African populations	Expand extended phenotyping and progressively introduce red-cell genotyping at reference centres
Antibody history	Historical antibodies may become undetectable	Maintain lifelong transfusion and antibody records and consider historical antibodies during unit selection
Transfusion reactions	Acute and delayed haemolytic reactions, including hyperhaemolysis	Establish standardized recognition, investigation, reporting, and specialist management pathways
Chronic transfusion	Cumulative transfusion exposure and iron overload	Use evidence-based indications, monitor iron burden, and provide appropriate chelation
Exchange transfusion	Limited access to automated apheresis and skilled personnel	Develop regional exchange-transfusion centres and resource-adapted manual exchange protocols
Laboratory capacity	Limited immunohaematology expertise, reagents, and infrastructure	Strengthen regional reference laboratories, quality systems, training, and specialist support
Haemovigilance	Under-reporting and limited transfusion-outcome surveillance	Establish integrated national/regional haemovigilance and adverse-event reporting systems
Patient identification and records	Fragmented transfusion histories and loss of antibody information	Develop longitudinal electronic transfusion records linked to SCD programmes and blood services
Equitable access	Geographic and socioeconomic barriers to specialist transfusion care	Use hub-and-spoke services, referral networks, telemedicine, and decentralized basic transfusion capacity
System sustainability	Dependence on emergency donation and fragmented services	Integrate SCD care with national blood-service planning, sustainable financing, workforce development, and regional collaboration

SCD, sickle cell disease; ABO, ABO blood-group system; RhD, Rhesus D; RH, Rhesus blood-group genes; HBV, hepatitis B virus; HCV, hepatitis C virus.

#### 4.7.1 Challenges in blood collection and storage and proposed solutions

Reliable blood collection and storage constitute the foundation of safe and sustainable haemotherapy for SCD in Africa. The effectiveness of transfusion medicine depends not only on whether blood can be obtained, but also on whether sufficient quantities of appropriately screened, processed, stored, transported, and traceable blood components remain available when patients require them. This distinction is particularly important for SCD because some patients require urgent transfusion during life-threatening complications, whereas others require predictable, repeated transfusion over months or years. Consequently, weaknesses in blood collection and storage can have both immediate clinical consequences and cumulative effects on long-term transfusion viability. One of the persistent challenges is the inability of some blood services to maintain a consistently adequate donor pool. Blood demand is generated by multiple clinical conditions, including obstetric haemorrhage, trauma, surgery, malaria-associated anaemia, cancer, and SCD. Consequently, SCD patients are managed within blood systems in which demand may frequently exceed collection capacity. Dependence on emergency or replacement donation can further destabilise supply. Replacement donation may temporarily address an individual patient's need but does not necessarily create a predictable, sustainable blood inventory. Seasonal variation, holidays, school closures, epidemics, population mobility, and public health emergencies can also substantially influence donor attendance. The solution should involve a transition toward stable voluntary, non-remunerated repeat donation systems supported by coordinated national and regional donor-recruitment programmes. Blood services should develop donor-retention strategies rather than focusing exclusively on one-time recruitment. Universities, workplaces, professional organisations, community groups, and other established social networks can provide platforms for regular donation campaigns. Public education should emphasise the importance of repeat donation and explain how sustained donation supports patients requiring chronic transfusion, including those with SCD (33).

The long-term viability of African blood services depends on developing a new generation of regular donors. Young adults represent an important potential donor population, yet recruitment programmes may be inconsistent or concentrated around specific institutions or campaigns. A sustainable strategy should therefore establish structured donor education and recruitment programmes within universities, colleges, workplaces, and communities, accompanied by appropriate donor eligibility assessment and protection of donor confidentiality. Digital communication platforms can facilitate appointment scheduling, reminders, donor education, and retention. The objective should not simply be to increase the number of registered donors, but to develop a reliable cohort of repeat donors capable of supporting predictable blood demand throughout the year. Blood collection may be concentrated in major urban centres, whereas many patients live in rural or peri-urban communities. Long travel distances, limited transportation infrastructure, and uneven distribution of blood-collection facilities can restrict access to donation opportunities and complicate movement of blood to areas of need. Mobile blood-collection teams can help address this geographical imbalance when supported by appropriate equipment, trained personnel, validated procedures, and reliable transportation. Strategic collection points can also be established in high-population areas and linked to central processing facilities. However, decentralisation should not occur without adequate quality assurance. Collection sites should operate within clearly defined standards for donor assessment, phlebotomy, specimen handling, cold-chain maintenance, documentation, and transportation (34).

Blood inventories can decline during predictable periods such as school holidays, major public holidays, examination periods, severe weather events, or seasonal infectious-disease outbreaks. These fluctuations can be particularly problematic for patients enrolled in chronic transfusion programmes because their transfusion schedules are less flexible than those of some other clinical groups. Blood services should therefore adopt seasonal inventory forecasting and proactive donor mobilisation. Historical collection and utilisation data can be used to identify predictable periods of shortage. Donor campaigns should begin before anticipated shortages rather than after inventory has already reached critically low levels. For SCD services, coordination between haematology clinics and blood centres can allow anticipated chronic transfusion requirements to be incorporated into inventory planning. Blood shortages can coexist with wastage when collection, distribution, and utilisation are poorly coordinated. Expiry of components, inappropriate ordering, inefficient stock rotation, inadequate temperature control, and failure to redistribute units between facilities can reduce the effective availability of blood. The first-expiry-first-out principle should be consistently applied, supported by accurate inventory records. Blood centres and hospitals should monitor collection, utilisation, wastage, expiry, and emergency-release patterns. Digital inventory-management systems can improve visibility of available units and facilitate redistribution between institutions. At a regional level, linked inventories could allow a unit approaching expiry in one location to be redirected to a facility with greater immediate demand, provided transport and storage requirements can be maintained (35).

For SCD, the quantity of blood in an inventory is only one component of availability. Patients who have developed alloantibodies may require antigen-negative units that are considerably more difficult to locate. A blood bank can therefore appear adequately stocked while being unable to provide a clinically suitable unit for a particular patient. This problem requires a transition from conventional inventory management toward compatibility-informed inventory management. Donor databases should progressively incorporate extended red-cell antigen information, while patients receiving repeated transfusions should have their clinically relevant antigen profiles and antibody histories documented. Regional or national rare-donor registries can further strengthen this system by identifying donors with uncommon antigen combinations. Where an appropriate unit is not available locally, predefined referral and transportation pathways can facilitate access from another blood centre (36). Collection is only the first stage of blood-component production. Blood must subsequently undergo appropriate processing, testing, component preparation, labelling, storage, and distribution. Limited component-processing capacity can restrict the ability of services to provide the most appropriate blood component for individual clinical indications. Strengthening component preparation requires investment in validated equipment, trained personnel, quality-control systems, appropriate storage facilities, and reliable maintenance. Where centralised processing is more efficient, regional hubs can provide component preparation for surrounding hospitals. Such centralisation should, however, be accompanied by dependable transportation and communication systems to ensure that processed components reach peripheral facilities within appropriate timeframes (33).

Maintaining the required storage conditions is fundamental to blood safety. Electricity interruptions, inadequate refrigeration, insufficient backup power, equipment failure, and transportation delays can compromise blood-component integrity and result in avoidable wastage. Blood establishments should develop resilient cold-chain systems that include validated refrigerators and freezers, continuous or appropriately frequent temperature monitoring, alarm systems, preventive maintenance, backup generators or other reliable power sources, and emergency contingency plans. The importance of this infrastructure extends beyond major blood centres. Peripheral hospitals that store blood must also maintain appropriate equipment and monitoring systems. Investments in solar-supported or other resilient power solutions may be particularly valuable in locations where grid reliability is limited, provided that systems are appropriately validated and maintained. The geographical distribution of African populations creates substantial transportation challenges. Blood collected in one location may need to be processed centrally and subsequently distributed to distant hospitals. Poor road infrastructure, long travel times, extreme temperatures, and inadequate transport containers can threaten component quality and availability. Blood transportation should therefore use validated temperature-controlled systems and documented transport procedures. Where feasible, regional distribution hubs can reduce transportation distances while maintaining quality assurance. Emergency transport pathways should also be established for patients with SCD requiring rare or specially matched units. Such systems are particularly important for patients with multiple alloantibodies, for whom locating compatible blood may take considerably longer than obtaining routinely compatible units (34).

Blood collection and storage systems are vulnerable to interruptions in the supply of reagents, blood bags, testing kits, filters, anticoagulants, calibration materials, and other consumables. Equipment may also become unusable because of delayed maintenance, unavailable spare parts, or insufficient technical support. Blood services should therefore implement strategic procurement and minimum-stock policies for critical consumables. Procurement planning should account for consumption rates, lead times, seasonal demand, and emergency reserves. Equipment procurement should similarly include lifecycle considerations. Purchasing a sophisticated device without ensuring access to maintenance, calibration, spare parts, software support, and trained technical personnel can produce long periods of operational downtime. Blood collection and storage require competent personnel across multiple disciplines, including phlebotomy, transfusion science, quality management, logistics, equipment maintenance, and clinical transfusion practice. Workforce shortages can increase workload and create vulnerabilities in donor assessment, blood processing, documentation, and inventory control. Investment in workforce development should include competency-based training, continuing professional development, supervision, competency assessment, and retention strategies. Cross-training can provide additional resilience during periods of staff absence or increased demand. Regional training centres may help standardise competencies and provide continuing education in blood collection, component processing, cold-chain management, immunohaematology, and haemovigilance (35).

Blood safety cannot be guaranteed through infectious-disease screening alone. Quality must be maintained from donor recruitment through final administration. Pre-analytical errors during donor registration or specimen collection can compromise otherwise accurate laboratory testing, while documentation errors can affect traceability. A comprehensive quality-management system should therefore include standard operating procedures, internal quality control, external quality assessment where applicable, equipment validation, temperature monitoring, staff competency assessment, documentation, non-conformance reporting, and corrective and preventive action. Regular audits should examine not only laboratory performance but also donor selection, collection practices, storage, transportation, inventory management, and traceability. Digital health technologies offer significant opportunities to improve blood availability and reduce wastage. Electronic donor registries can support donor recruitment, eligibility tracking, appointment scheduling, and repeat-donation reminders. Electronic inventory systems can provide real-time information about blood groups, component types, expiry dates, locations, and utilisation patterns. For SCD, digitalisation can be particularly valuable when linked to patient transfusion records. An integrated system could allow authorised blood-service personnel to view a patient's historical blood group, extended phenotype or genotype, alloantibody history, previous transfusion reactions, and special blood requirements. Implementation should be phased according to infrastructure and financial capacity. Where fully electronic systems are not immediately feasible, standardised paper-based or hybrid systems can provide an interim approach (36).

SCD services have a distinctive advantage for blood planning because some transfusion requirements are predictable. Patients enrolled in chronic transfusion programmes generally have scheduled transfusions, allowing blood services to anticipate at least part of their demand. Haematology clinics and blood centres should therefore establish routine communication mechanisms for sharing projected transfusion requirements. This approach can reduce emergency requests and improve preparation of appropriately matched units. Demand forecasting should also account for patients with recurrent acute complications and those undergoing planned surgery. Early notification of anticipated transfusion needs allows blood services additional time to identify antigen-negative or otherwise specially selected units. Blood collection and storage require continuous financing rather than episodic investment. Costs include donor recruitment, collection equipment, blood bags, infectious-disease screening, refrigeration, transportation, quality assurance, workforce development, information systems, and equipment maintenance. Sustainable financing models should therefore be incorporated into national health planning. Investment in blood services should be regarded as an essential component of SCD care and broader emergency health-system capacity rather than as an isolated laboratory expense. Economic planning should also account for the costs of blood wastage, emergency procurement, delayed transfusion, transfusion reactions, and complications arising from inadequate compatibility. Investments that improve inventory efficiency and reduce avoidable complications may therefore generate substantial downstream health-system benefits (33).

#### 4.7.2 Proposed integrated solution

The challenges of blood collection and storage cannot be solved through a single intervention. A coordinated strategy should establish a resilient blood-supply architecture based on six interconnected pillars:

1. Sustainable donor base: Expand voluntary repeat donation through structured community and institutional recruitment.
2. Intelligent collection and inventory management: Use demand forecasting, appropriate stock rotation, real-time inventory monitoring, and inter-facility redistribution to reduce shortages and wastage.
3. Resilient infrastructure: Strengthen cold-chain systems, backup power, transportation, equipment maintenance, and critical consumable procurement.
4. Compatibility-informed supply: Integrate extended donor antigen typing, patient antigen profiles, antibody histories, and rare-donor registries into inventory planning.
5. Regional integration: Establish networks linking blood centres, hospitals, reference laboratories, and specialised SCD services.
6. Digital and quality infrastructure: Develop traceable electronic systems, standardised documentation, quality-management programmes, and haemovigilance mechanisms.

### 4.8 Blood availability and supply dynamics

Reliable access to safe blood is the rate-limiting step for effective hemotherapy in sickle cell disease (SCD) across much of Africa. Supply is shaped by the size and stability of the donor pool, the organization of blood services, and the integrity of collection, processing, and distribution systems. In practice, these elements interact to produce marked variability in availability—between countries, within regions, and even across facilities in the same city—directly influencing clinical decision-making and outcomes (37). Donation rates in many settings remain below levels required to meet aggregate clinical demand, with SCD adding a steady, non-seasonal requirement on top of obstetric, trauma, and pediatric indications. A persistent structural feature is reliance on family replacement donors, which can generate blood at the point of need but yields an unstable supply and complicates inventory planning. By contrast, repeat voluntary non-remunerated donors provide a more predictable and safer donor base, yet their proportion remains suboptimal in several countries due to sociocultural barriers, limited awareness, and weak retention systems. Effective recruitment strategies are those that convert first-time donors into regular donors through targeted communication, donor-friendly services, and feedback loops (e.g., reminders, recognition programs). Engagement of schools, universities, workplaces, and faith-based organizations has proven particularly productive where sustained partnerships are maintained (38, 39).

The architecture of blood services influences both availability and equity. Fragmented, hospital-based collection models often struggle with quality standardization and economies of scale. Regionalized or nationally coordinated blood transfusion services—centralizing testing and component preparation while decentralizing collection through mobile teams—tend to improve efficiency, quality assurance, and stock visibility. However, centralization must be matched with dependable distribution networks; otherwise, peripheral facilities experience stock-outs despite adequate national collections. Investments in transport logistics, route optimization, and real-time communication between hubs and hospitals are therefore pivotal (40). Infrastructure constraints remain a critical determinant of supply stability. Cold chain integrity—from collection to transfusion—is vulnerable to intermittent power supply, limited refrigeration capacity, and equipment downtime. Backup power solutions, temperature monitoring, and preventive maintenance protocols are essential to reduce wastage and ensure product quality. Laboratory capacity for component separation is uneven; as a result, whole blood is still widely used. Expanding component therapy allows a single donation to meet multiple clinical needs and can improve overall system efficiency, but requires capital investment, trained personnel, and consistent consumable supply (41, 42).

Inventory management practices directly affect both availability and wastage. Facilities that rely on manual stock tracking often lack visibility of near-expiry units or shortages across the network. Simple digital inventory systems—ranging from spreadsheet-based tools to dedicated blood bank software—enable forecasting, redistribution between sites, and prioritization for high-need patients such as those on chronic transfusion programs. Establishing minimum stock thresholds and emergency buffer stocks for SCD-heavy centers can mitigate acute shortages. In parallel, clear clinical prioritization frameworks are necessary when supply is constrained, ensuring that life-threatening indications (e.g., severe anemia, acute chest syndrome, stroke) are met first (43). Geographic inequities are pronounced. Rural and peripheral facilities frequently face longer resupply times, fewer storage capabilities, and limited testing services. Outreach collection, satellite storage points with validated cold chain, and scheduled distribution cycles can narrow these gaps. Telecommunication links between peripheral sites and regional hubs facilitate rapid requisition and clinical guidance, particularly during emergencies (44). Seasonality further complicates supply. School holidays, agricultural cycles, and public health emergencies can reduce donor turnout, while periods of increased disease burden elevate demand. Anticipatory planning—intensifying donor drives before predictable downturns and building buffer stocks—helps smooth these fluctuations. Public communication that aligns donation campaigns with these cycles is more effective than *ad hoc* appeals (45).

Financial structures also influence availability. Where patients bear direct costs for blood or processing, demand may be suppressed or delayed, distorting planning and exacerbating inequities. Incorporation of blood services into publicly financed packages or insurance schemes stabilizes demand and supports more rational procurement and distribution. At the system level, pooled procurement of reagents and consumables reduces stock-outs and unit costs (44). For SCD specifically, demand management is as important as supply expansion. Protocolized transfusion practices, integration of hydroxyurea to reduce transfusion frequency, and appropriate use of exchange transfusion (where feasible) can optimize utilization of scarce blood. Dedicated scheduling for patients on chronic transfusion programs improves predictability, enabling better inventory planning and reducing emergency dependence (Figure 1) (45).

**Figure 1:**
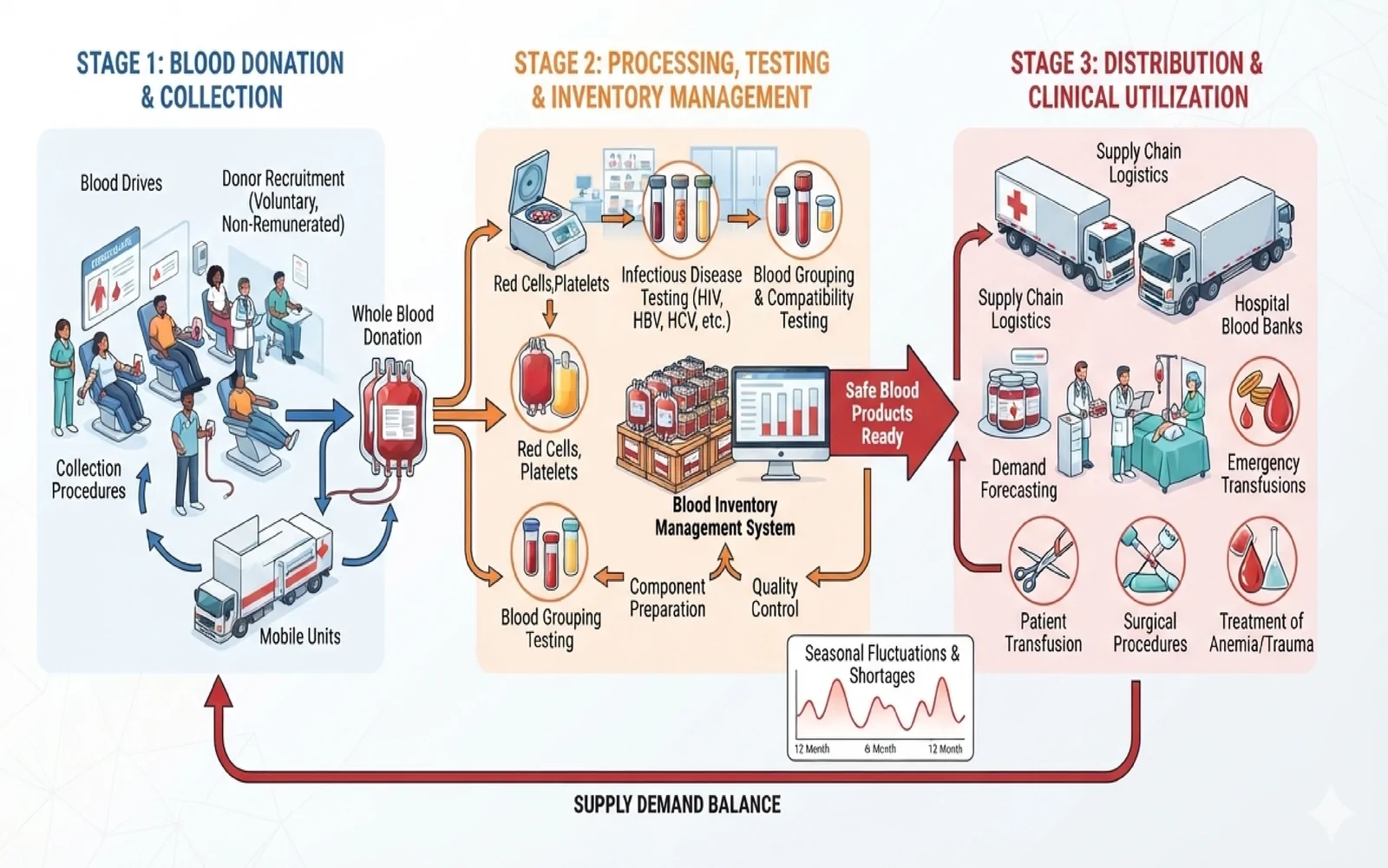
Blood availability and supply dynamics.

### 4.9 Long-Term sustainability of hemotherapy services

Sustaining hemotherapy for SCD in African health systems requires more than intermittent improvements in supply; it depends on durable financing, institutionalized governance, a competent workforce, resilient infrastructure, and continuous quality management. Because transfusion support in SCD is often recurrent—particularly for stroke prevention and other high-risk phenotypes—programs must be designed to function reliably over years, not just respond to episodic demand (36). A defining constraint on sustainability is the fragmentation of funding streams for blood services. Where costs of collection, testing, and distribution are partially or wholly passed to patients, access becomes inequitable and utilization unpredictable. Embedding blood transfusion services within national health financing frameworks—whether tax-funded systems or social health insurance—stabilizes demand and enables forward planning. Ring-fenced budget lines for national blood programs, coupled with pooled procurement of reagents and consumables, reduce unit costs and mitigate stock-outs. For SCD specifically, inclusion of transfusion (and iron chelation where indicated) in essential benefits packages prevents interruption of chronic programs and reduces downstream complications that are more expensive to treat (37, 38).

Strong stewardship is associated with more consistent quality and supply. National blood policies that define standards for donor recruitment, testing algorithms, component preparation, and distribution create a common operating framework across public and private providers. Regulatory oversight—licensure of facilities, proficiency testing, and periodic audits—helps maintain adherence to standards. Importantly, governance should also formalize hemovigilance, mandating reporting of adverse events and creating feedback loops that inform practice. Alignment with international norms, adapted to local capacity, promotes incremental quality gains without imposing infeasible requirements (39). Sustainable models typically balance centralized and decentralized functions. Centralized testing and component preparation support quality assurance and economies of scale, while decentralized collection and clinical interfaces maintain proximity to donors and patients. Integration with broader health services is essential: antenatal clinics, HIV programs, schools, and workplaces can serve as stable platforms for donor recruitment; SCD clinics can be linked to scheduled transfusion services, improving predictability and inventory planning. Cross-border or regional collaboration may be advantageous for smaller countries, enabling shared reference laboratories and procurement mechanisms (40).

Human resources are a recurrent bottleneck. Sustainable programs require a pipeline of trained professionals in transfusion medicine, laboratory sciences, and clinical hematology, alongside nurses and technicians skilled in blood administration and monitoring. Competency-based training, standardized protocols, and continuous professional development reduce variability in practice. Task-shifting—supported by clear guidelines and supervision—can expand service coverage in settings with limited specialist availability. Retention strategies, including career progression pathways and supportive work environments, are necessary to prevent attrition (41, 42). Reliable hemotherapy depends on robust cold chain systems, functional laboratories, and dependable transport networks. Investments in energy resilience (e.g., backup power, solar solutions), equipment maintenance, and temperature monitoring reduce wastage and protect product quality. Digital tools for inventory management and distribution planning enhance visibility across the network, enabling redistribution before expiry and rapid response to shortages. Local or regional maintenance capacity for equipment and secured supply chains for reagents are critical to avoid service interruptions (42).

Sustainability is closely linked to the institutionalization of quality management systems. Standard operating procedures, internal quality control, and participation in external quality assessment schemes support consistent performance. Hemovigilance systems—adapted to local context—enable detection of adverse events, trend analysis, and targeted corrective actions. Even simple, standardized reporting mechanisms can drive meaningful improvements when coupled with regular review and accountability (43). Selective, context-appropriate adoption of technology can improve efficiency and outcomes. Examples include digital donor registries to enhance retention, barcoding for traceability, and low-cost apheresis options to expand exchange transfusion capacity. However, sustainability hinges on total cost of ownership—procurement, maintenance, consumables, and training—rather than acquisition alone. Technologies should be introduced with clear use-cases, interoperability with existing systems, and plans for local support (44).

Sustainable blood systems align supply with rational use. In SCD, protocolized transfusion indications, integration of disease-modifying therapy (e.g., hydroxyurea) to reduce transfusion frequency, and prioritization frameworks during shortages help preserve limited resources. Structured chronic transfusion programs—with scheduled appointments and predefined targets—convert unpredictable demand into planned utilization, improving both patient outcomes and inventory control (45). External partners have played an important role in strengthening blood services, but long-term sustainability requires progressive transition to domestic financing and governance. Partnerships should be structured to build local capacity—technical, managerial, and regulatory—while avoiding parallel systems. Transparent metrics and milestones facilitate this transition (44). Sustainability depends on data. Routine indicators—donation rates, proportion of voluntary repeat donors, unit wastage, stock-out frequency, TTI prevalence among donors, alloimmunization rates in SCD cohorts, and adverse event reporting—should inform management decisions. Public reporting and performance benchmarking can reinforce accountability and drive continuous improvement (Figure 2) (45).

**Figure 2:**
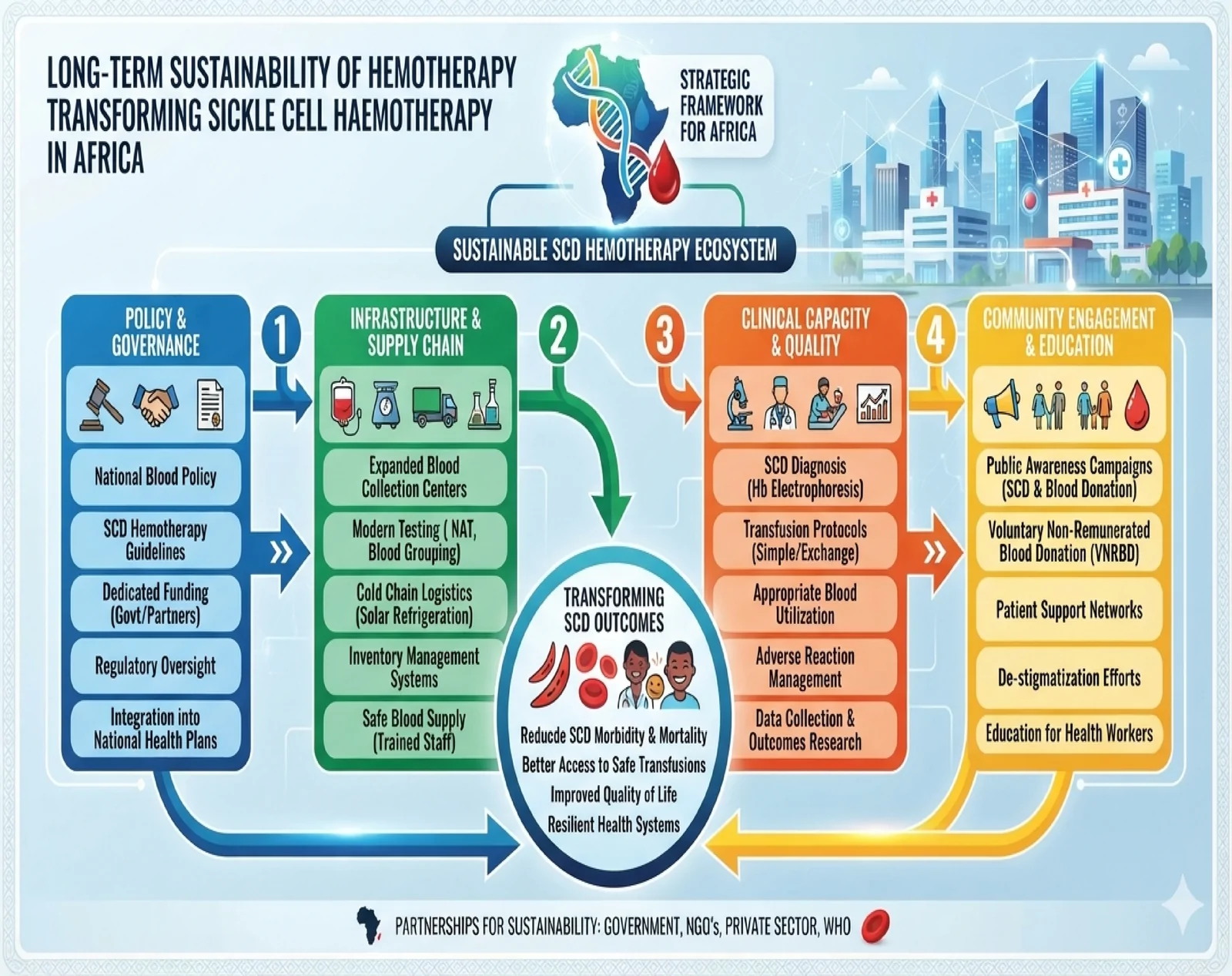
Long-Term sustainability of hemotherapy services.

### 4.10 Recommendations

The evidence reviewed indicates that improving haemotherapy for SCD in Africa requires a coordinated shift from fragmented, reactive transfusion practices toward integrated, patient-centred, and sustainable haemotherapeutic systems**.** The following recommendations are proposed to address blood availability, transfusion safety, immunohaematological compatibility, haemovigilance, iron management, and long-term system resilience.

#### 4.10.1 Strengthen voluntary repeat blood donation

National blood services should prioritise sustained voluntary, non-remunerated blood donation rather than dependence on emergency or replacement donation. Donor recruitment should be embedded within universities, workplaces, communities, professional organisations, and other established social structures. Particular emphasis should be placed on donor retention, repeat donation, donor education, and year-round mobilisation to reduce seasonal shortages.

#### 4.10.2 Develop SCD-informed blood demand forecasting

Blood services should incorporate the predictable requirements of patients receiving chronic transfusion into national and regional inventory planning. Haematology units should communicate anticipated transfusion schedules to blood centres, allowing compatible units to be identified before they are urgently required. Demand forecasting should also incorporate anticipated surgical procedures and patients with recurrent severe complications.

#### 4.10.3 Move from blood availability to compatible blood availability

Blood-service performance should not be measured solely by the number of units collected. Particular attention should be given to whether appropriately compatible units are available for individual SCD patients. Donor databases should progressively incorporate extended red-cell antigen information, while patients receiving repeated transfusions should have their clinically relevant antigen profiles documented.

#### 4.10.4 Establish risk-based extended antigen matching

ABO and RhD compatibility should remain fundamental, but frequently transfused SCD patients should receive more comprehensive antigen matching where resources permit. Rh and Kell matching should form an important foundation, while Duffy, Kidd, MNS, and other clinically significant antigens should be considered according to the patient's antibody history, transfusion burden, antigen profile, and local blood-service capacity. This risk-based approach is preferable to a uniform strategy that may be difficult to implement in settings with constrained inventories.

#### 4.10.5 Expand molecular red-cell genotyping selectively

Molecular genotyping should be progressively incorporated for patients with complex Rh variants, multiple alloantibodies, serological discrepancies, recent transfusion that compromises conventional phenotyping, or difficulty identifying compatible blood. Regional reference laboratories could provide this service where individual hospitals cannot sustain molecular testing.

#### 4.10.6 Establish lifelong transfusion and antibody records

Every patient receiving recurrent transfusion should have a durable transfusion record containing ABO/RhD status, extended phenotype or genotype where available, antibody history, transfusion exposure, documented reactions, and special compatibility requirements. Where interoperable electronic health systems are unavailable, standardised patient-held transfusion records should provide an interim solution. Historical antibody information should remain permanently accessible**,** even when an antibody is no longer detectable.

#### 4.10.7 Establish regional immunohaematology reference laboratories

Countries and regional health networks should develop reference laboratories capable of antibody identification, complex compatibility testing, investigation of haemolytic transfusion reactions, red-cell genotyping, and support for patients with rare phenotypes or multiple alloantibodies. Centralising highly specialised services can be more sustainable than attempting to provide advanced immunohaematology in every hospital.

#### 4.10.8 Develop national and regional rare-donor registries

Rare-donor registries linked to extended antigen typing should be established to improve access to compatible units for patients with uncommon phenotypes or multiple antibodies. Regional collaboration should allow appropriately screened units to be located and transferred across institutional or national boundaries when necessary.

#### 4.10.9 Strengthen transfusion-transmitted infection prevention

Blood services should maintain rigorous donor selection and validated screening for locally relevant transfusion-transmitted infections. Quality assurance should encompass specimen identification, assay performance, reagent management, internal quality control, external quality assessment, equipment maintenance, and appropriate management of reactive donations. Where advanced molecular testing is introduced, it should complement rather than substitute for strong foundational quality systems.

#### 4.10.10 Improve cold-chain resilience and blood storage

Investment should prioritise validated refrigeration and storage systems, continuous temperature monitoring, backup power, preventive maintenance, and emergency contingency plans. Transportation systems should maintain appropriate environmental conditions and provide documented traceability from blood collection through final use. Solar-supported or other resilient energy systems may be considered in locations with unreliable electricity, provided they meet validated storage requirements.

#### 4.10.11 Reduce blood wastage through intelligent inventory management

Blood centres should systematically monitor collection, utilisation, expiry, wastage, emergency-release practices, and stock levels. First-expiry-first-out inventory rotation should be consistently implemented. Digital inventory systems should progressively provide real-time visibility of blood groups, component types, expiry dates, and locations. Regional redistribution mechanisms can help transfer units from facilities with excess inventory to those experiencing shortages.

#### 4.10.12 Strengthen haemovigilance

Functional haemovigilance systems should be established or strengthened at institutional, regional, and national levels. These systems should capture acute and delayed transfusion reactions, alloimmunisation, hyperhaemolysis, transfusion-transmitted infections, identification errors, and other significant adverse events. Reporting should operate within a non-punitive safety culture that encourages transparency and learning. Significant events should undergo structured investigation and, where appropriate, root-cause analysis followed by corrective and preventive action.

#### 4.10.13 Integrate iron surveillance into chronic transfusion programmes

Patients receiving repeated transfusions should undergo systematic assessment of cumulative iron burden. Serum ferritin trends can support longitudinal monitoring, while validated assessment of tissue iron should be considered where available and clinically appropriate. Chelation therapy should be accessible to patients who require it, with attention to adherence, adverse effects, treatment response, and appropriate monitoring. Red-cell exchange may be considered for selected patients where its clinical benefits and resource requirements are justified.

#### 4.10.14 Expand patient blood management

Patient Blood Management should become an integral component of SCD care. The strategy should include appropriate transfusion thresholds, optimisation of endogenous erythropoiesis, prevention of avoidable blood loss, rational use of blood components, and reduction of unnecessary transfusion exposure. The objective should not be simply to reduce transfusion numbers but to ensure that transfusion is provided when its expected clinical benefit clearly outweighs cumulative risk.

#### 4.10.15 Strengthen access to disease-modifying therapies

Improving haemotherapy must occur alongside expansion of disease-modifying SCD care. Hydroxyurea and other evidence-based therapies should be made more accessible, with appropriate laboratory monitoring and adherence support. Effective disease modification can reduce vaso-occlusive complications and other events that generate preventable transfusion requirements, thereby benefiting both individual patients and constrained blood systems.

#### 4.10.16 Expand access to red-cell exchange where clinically justified

Red-cell exchange should be developed progressively at appropriately selected tertiary or regional centres for severe acute complications and chronic indications where it provides a clear clinical advantage. Implementation should be accompanied by investment in equipment, trained personnel, vascular-access expertise, compatible blood supply, maintenance systems, and appropriate clinical governance. It should not be assumed that every hospital requires an exchange programme; regional centres may provide a more sustainable model.

#### 4.10.17 Develop integrated digital transfusion infrastructure

Electronic blood-bank and clinical information systems should progressively incorporate donor information, blood inventory, patient antigen profiles, antibody histories, transfusion episodes, and adverse reactions. Interoperability between blood centres and hospitals should be prioritised. Where comprehensive digitalisation is not immediately feasible, standardised hybrid systems should be implemented to ensure continuity of essential transfusion information.

#### 4.10.18 Strengthen workforce capacity

African blood services should invest in competency-based training and continuing professional development for medical laboratory scientists, transfusion specialists, clinicians, nurses, blood-bank personnel, quality managers, and biomedical engineers. Training should include SCD-specific transfusion practice, immunohaematology, antigen matching, transfusion-reaction investigation, haemovigilance, quality management, inventory management, and cold-chain maintenance. Workforce retention should receive equal attention to initial training.

#### 4.10.19 Strengthen quality management and regulatory oversight

National blood services and hospital transfusion departments should implement comprehensive quality-management systems encompassing standard operating procedures, equipment validation, internal quality control, external quality assessment, staff competency assessment, documentation, audits, non-conformance management, and corrective and preventive action. Regulatory authorities should ensure consistent application of national blood-safety standards and strengthen accreditation and inspection mechanisms.

#### 4.10.20 Develop sustainable financing mechanisms

Blood safety requires continuous rather than intermittent investment. National health systems should provide protected funding for donor recruitment, infectious-disease screening, blood bags and reagents, refrigeration, transportation, equipment maintenance, workforce development, information systems, haemovigilance, and specialised immunohaematology. Investment should be evaluated in relation to the downstream costs of transfusion delays, blood wastage, alloimmunisation, iron overload, transfusion reactions, and avoidable hospitalisation.

#### 4.10.21 Develop African-specific evidence and implementation research

Further research should evaluate the effectiveness, cost-effectiveness, and feasibility of different transfusion strategies within African populations. Priority areas include optimal antigen-matching approaches, molecular blood-group variation, rare-donor networks, red-cell exchange, digital transfusion records, iron-monitoring strategies, donor-retention interventions, and regional blood-sharing systems. Importantly, future research should move beyond documenting the prevalence of transfusion complications toward testing implementable solutions and measurable health-system outcomes**.**

#### 4.10.22 Proposed implementation framework

A phased implementation strategy may provide the most realistic pathway:

##### 4.10.22.1 Phase I – Foundation

Strengthen voluntary donation, infectious-disease screening, cold-chain systems, basic quality management, transfusion documentation, and reliable inventory control.

##### 4.10.22.2 Phase II – Compatibility and surveillance

Introduce extended antigen profiling for frequently transfused SCD patients, risk-based Rh/Kell matching, comprehensive antibody documentation, haemovigilance, iron monitoring, and regional immunohaematology referral systems.

##### 4.10.22.3 Phase III – Advanced haemotherapeutic capacity

Expand molecular genotyping, rare-donor registries, interoperable digital transfusion systems, red-cell exchange services, and regional compatible-blood networks.

##### 4.10.22.4 Phase IV – Sustainable integration

Integrate transfusion services with comprehensive SCD programmes, Patient Blood Management, disease-modifying therapies, national health financing, and continuous quality-improvement systems.

## 5 Conclusion

Haemotherapy remains indispensable to the management of selected acute and chronic complications of SCD in Africa, but its effectiveness is determined by the strength of the blood-transfusion system within which it is delivered. The central challenge is therefore not simply increasing the quantity of available blood, but ensuring sustained access to safe, compatible, traceable, clinically appropriate, and affordable blood throughout the patient's lifetime**.** Blood shortages, variable transfusion-service capacity, transfusion-transmitted infections, alloimmunisation, complex red-cell antigen profiles, iron overload, fragmented transfusion histories, and inadequate haemovigilance remain interconnected threats to optimal SCD care. A sustainable African haemotherapeutic pathway should move beyond episodic transfusion toward an integrated model encompassing voluntary donor recruitment, robust infectious-disease screening, reliable inventory management, extended red-cell antigen profiling, risk-based antigen matching, molecular genotyping for selected complex cases, lifelong antibody documentation, regional immunohaematology reference services, rare-donor registries, systematic haemovigilance, and longitudinal iron monitoring. Rh and Kell matching should provide an important foundation for frequently transfused patients, while Duffy, Kidd, MNS, and other clinically significant antigens should be incorporated according to individual antibody history, transfusion burden, population characteristics, and local laboratory capacity.

Equally important, transfusion services should be integrated with comprehensive SCD care rather than functioning as isolated blood-supply mechanisms. Hydroxyurea and other disease-modifying therapies, infection prevention, stroke surveillance, patient blood management, and appropriate clinical monitoring can reduce preventable complications and unnecessary transfusion exposure. Where resources permit, red-cell exchange may provide an important strategy for selected patients requiring substantial HbS reduction while limiting net iron accumulation. The future of SCD haemotherapy in Africa therefore depends on transfusion-system resilience rather than transfusion volume alone**.** Investment should prioritise interoperable transfusion information systems, trained personnel, quality-management systems, regional blood-sharing networks, sustainable financing, and equitable access to specialised immunohaematology. Such investments can convert fragmented transfusion services into coordinated lifelong haemotherapeutic pathways.

## References

[1] Taher M Aminondin S', Nasir NA Jasmadi NA Nizam NIN Shahrul IS. Sickle cell disease: understanding pathophysiology, clinical features and advances in gene therapy approaches. Front Pharmacol. (2025) 16:1630994. 10.3389/fphar.2025.1630994 40918513 PMC12411783

[2] Obeagu EI Ubosi NI Obeagu GU Egba SI Bluth MH. Understanding apoptosis in sickle cell anemia patients: mechanisms and implications. Medicine (Baltimore). (2024) 103(2):e36898. 10.1097/MD.0000000000036898 38215146 PMC10783340

[3] Makani J Ofori-Acquah SF Nnodu O Wonkam A Ohene-Frempong K. Sickle cell disease: new opportunities and challenges in Africa. ScientificWorldJournal. (2013) 2013:193252. 10.1155/2013/193252 25143960 PMC3988892

[4] Howard J. Sickle cell disease: when and how to transfuse. Hematology Am Soc Hematol Educ Program. (2016) 2016(1):625–31. 10.1182/asheducation-2016.1.625 27913538 PMC6142434

[5] Alsharari E Enazi A Hanafy A Mustafa E Abufara M Altoonisi F. Indications for blood transfusion and exchange transfusion in sickle cell disease: a single center experience. Cureus. (2025) 17(1):e77499. 10.7759/cureus.77499 39958095 PMC11828706

[6] Bloch EM Vermeulen M Murphy E. Blood transfusion safety in Africa: a literature review of infectious disease and organizational challenges. Transfus Med Rev. (2012) 26(2):164–80. 10.1016/j.tmrv.2011.07.006 21872426 PMC3668661

[7] Djientcheu Deugoue FY Azabji-Kenfack M Tayou Tagny C Kouamou Tchiekou MA Menteng Tchuente SA Deneys V. The issue of screening for transfusion-transmitted infections in Sub-Saharan Africa presents several challenges: a review. Adv Hematol. (2025) 2025:1373786. 10.1155/ah/1373786 41164286 PMC12562600

[8] Obeagu EI. Blood transfusion practices in Africa: advances, barriers, and ways of overcoming the barriers—a narrative review. Ann Med Surg (Lond). (2025) 87(11):7267–77. 10.1097/MS9.0000000000003898 PMC12577889 41180718

[9] Obeagu EI Alsadi RA. Blood banking systems in Africa: challenges, innovations, and recommendations for strengthening blood banking systems in Africa–A narrative review. Univers J Pharm Res. (2025) 10(3):52–8. 10.22270/ujpr.v10i3.1359

[10] Osaro E Charles AT. The challenges of meeting the blood transfusion requirements in Sub-Saharan Africa: the need for the development of alternatives to allogenic blood. J Blood Med. (2011) 2:7–21. 10.2147/JBM.S17194 22287859 PMC3262349

[11] Alemnji GA Zeh C Yao K Fonjungo PN. Strengthening national health laboratories in Sub-Saharan Africa: a decade of remarkable progress. Trop Med Int Health. (2014) 19(4):450–8. 10.1111/tmi.12269 24506521 PMC4826025

[12] Regalado-Artamendi I Pérez-Corral AM García-Morín M Cela E Beléndez C Bardón-Cancho EJ. Complete RH and kell matching related to low alloimmunisation risk in sickle cell disease: prevalence and risk factors of alloimmunisation in a Spanish tertiary care national reference centre. Blood Transfus. (2021) 19(4):292–9. 10.2450/2020.0096-20 33085598 PMC8297678

[13] Kanagasabai U Qualls M Shiraishi RW Eno L Zungu I Bust L. Baseline assessment findings of the Africa society for blood transfusion step-wise accreditation programme in 10 Sub-Saharan African countries, 2016−2018. Vox Sang. (2022) 117(6):839–46. 10.1111/vox.13262 35266552 PMC9314842

[14] Linder GE Chou ST. Red cell transfusion and alloimmunization in sickle cell disease. Haematologica. (2021) 106(7):1805–15. 10.3324/haematol.2020.270546 33792218 PMC8252926

[15] Han H Hensch L Tubman VN. Indications for transfusion in the management of sickle cell disease. Hematology Am Soc Hematol Educ Program. (2021) 2021(1):696–703. 10.1182/hematology.2021000307 34889416 PMC8791131

[16] Novelli EM Gladwin MT. Crises in sickle cell disease. Chest. (2016) 149(4):1082–93. 10.1016/j.chest.2015.12.016 26836899 PMC6637264

[17] Adjepong KO Otegbeye F Adjepong YA. Perioperative management of sickle cell disease. Mediterr J Hematol Infect Dis. (2018) 10(1):e2018032. 10.4084/MJHID.2018.032 29755709 PMC5937979

[18] Weimer A Tagny CT Tapko JB Gouws C Tobian AAR Ness PM. Blood transfusion safety in Sub-Saharan Africa: a literature review of changes and challenges in the 21st century. Transfusion. (2019) 59(1):412–27. 10.1111/trf.14949 30615810

[19] Aneke JC Okocha CE. Blood transfusion safety; current status and challenges in Nigeria. Asian J Transfus Sci. (2017) 11(1):1–5. 10.4103/0973-6247.200781 28316432 PMC5345273

[20] Jayaraman S Chalabi Z Perel P Guerriero C Roberts I. The risk of transfusion-transmitted infections in Sub-Saharan Africa. Transfusion. (2010) 50(2):433–42. 10.1111/j.1537-2995.2009.002402 19843290

[21] Sysol JR Machado R. Sickle cell disease and acute chest syndrome: epidemiology, diagnosis, management, outcomes. Hematologic Abnormalities and Acute Lung Syndromes. (2016):67–87.

[22] Puerto-Meredith S Singogo E Chagomerana M Nthani T Likaka A Gondwe A. Systematic review of prevalence and risk factors of transfusion transmissible infections among blood donors, and blood safety improvements in Southern Africa. Transfus Med. (2023) 33(5):355–71. 10.1111/tme.12988 37641525 PMC11070456

[23] Noizat-Pirenne F. Relevance of blood groups in transfusion of sickle cell disease patients. C R Biol. (2013) 336(3):152–8. 23643398 10.1016/j.crvi.2012.09.011

[24] Thein SL Pirenne F Fasano RM Habibi A Bartolucci P Chonat S. Hemolytic transfusion reactions in sickle cell disease: underappreciated and potentially fatal. Haematologica. (2020) 105(3):539–44. 10.3324/haematol.2019.224709 32029505 PMC7049330

[25] Mammen JJ Asirvatham ES Sarman CJ Ranjan V Charles B. A review of legal, regulatory, and policy aspects of blood transfusion services in India: issues, challenges, and opportunities. Asian J Transfus Sci. (2021) 15(2):204–11. 10.4103/ajts.AJTS_65_20 34908756 PMC8628249

[26] Aalaei S Amini S Keramati MR Shahraki H Eslami S. Monitoring of storage and transportation temperature conditions in red blood cell units: a cross-sectional study. Indian J Hematol Blood Transfus. (2019) 35(2):304–12. 10.1007/s12288-018-1038-6 30988568 PMC6439098

[27] Nyirimanzi JD Ngenzi J Kagisha V Bizimana T Kayitare E. Assessment of medicines cold chain storage conformity with the requirements of the world health organization in health facilities of the eastern province of Rwanda. J Pharm Policy Pract. (2023) 16(1):31. 10.1186/s40545-023-00534-3 36855183 PMC9974057

[28] Boukhris S Chouchene S Gaaloul M Mezrigui R Aissi RE Slama N. Are healthcare professionals adequately trained for safe blood transfusions? Targeted training to bridge knowledge gaps. Indian J Hematol Blood Transfus. (2024) 40(4):682–6. 10.1007/s12288-024-01778-y 39469183 PMC11512939

[29] Jacobs JW Amorim L Pirenne F Tayou C Adimora I Pecker LH. The wider perspective: barriers and recommendations for transfusion support for patients with sickle cell disease in low- and middle-income countries. Br J Haematol. (2025) 206(6):1585–92. 10.1111/bjh.20055 40147455 PMC12166344

[30] Odame I Bazuaye GN. Transfusions, disease-modifying treatments, and curative therapies for sickle cell anemia in Africa: where are we now? Hematology Am Soc Hematol Educ Program. (2024) 2024(1):234–9. 10.1182/hematology.2024000550 39643983 PMC11665607

[31] Vellanki S Thalambedu N Kumar AKTA Vellanki S Honhar M Hendrix R. Navigating hyperhemolysis in sickle cell disease: insights from literature. Diagnostics (Basel). (2025) 15(14):1835. 10.3390/diagnostics15141835 40722584 PMC12293273

[32] Mohammed AD Ntambwe P Crawford AM. Barriers to effective transfusion practices in limited-resource settings: from infrastructure to cultural beliefs. World J Surg. (2020) 44(7):2094–9. 10.1007/s00268-020-05461-x 32157404 PMC7266790

[33] Bates I Hassall O Mapako T. Transfusion research priorities for blood services in Sub-Saharan Africa. Br J Haematol. (2017) 177(6):855–63. 10.1111/bjh.14577 28449225

[34] Gasparovic Babic S Krsek A Baticic L. Voluntary blood donation in modern healthcare: trends, challenges, and opportunities. Epidemiologia (Basel). (2024) 5(4):770–84. 10.3390/epidemiologia5040052 39727424 PMC11675880

[35] Makin JK Francis KL Polonsky MJ Renzaho AMN. Interventions to increase blood donation among ethnic/racial minorities: a systematic review. J Environ Public Health. (2019) 2019:1–14. 10.1155/2019/6810959 PMC6501160 31143209

[36] Obeagu EI. Global awareness of antigenic peptides—unraveling the impact on sickle cell anemia patients in the presence of pathogens: a narrative review. Medicine (Baltimore). (2024) 103(36):e39513. 10.1097/MD.0000000000039513 39252321 PMC12431756

[37] Gammon RR Rosenbaum L Cooke R Friedman M Rockwood L Nichols T. Maintaining adequate donations and a sustainable blood supply: lessons learned. Transfusion. (2021) 61(1):294–302. 10.1111/trf.16145 33206404 PMC7753343

[38] Ilesanmi OS Afolabi AA. Sustainability of donor-funded health-related programs beyond the funding lifecycle in Africa: a systematic review. Cureus. (2022) 14(5):e24643. 10.7759/cureus.24643 35663674 PMC9156348

[39] Rambiritch V Vermeulen M Bell H Knox P Nedelcu E Al-Riyami AZ. Education subcommittee of the AABB global transfusion forum. Transfusion medicine and blood banking education and training for blood establishment laboratory staff: a review of selected countries in Africa. Transfusion. (2021) 61(6):1955–65. 10.1111/trf.16372 33738810 PMC8217161

[40] Samukange WT Kafere C Heinrich K Sabblah GT Siame MM Chirinda L. Strengthening blood regulatory systems to tackle Africa's Unmet needs for blood and blood products. Transfus Med Hemother. (2023) 50(2):123–8. 10.1159/000528077 37066057 PMC10090969

[41] Mahomed OH Asmall S Voce A. Sustainability of the integrated chronic disease management model at primary care clinics in South Africa. Afr J Prim Health Care Fam Med. (2016) 8(1):e1–7. 10.4102/phcfm.v8i1.1248 PMC5125260 28155314

[42] Molero A Calabrò M Vignes M Gouget B Gruson D. Sustainability in healthcare: perspectives and reflections regarding laboratory medicine. Ann Lab Med. (2021) 41(2):139–44. 10.3343/alm.2021.41.2.139 33063675 PMC7591295

[43] Faizan M Han C Lee SW. Policy-Driven digital health interventions for health promotion and disease prevention: a systematic review of clinical and environmental outcomes. Healthcare (Basel). (2025) 13(18):2319. 10.3390/healthcare13182319 41008449 PMC12469472

[44] Pruett CR Vermeulen M Zacharias P Ingram C Tagny T Bloch C. The use of rapid diagnostic tests for transfusion infectious screening in Africa: a literature review. Transfus Med Rev. (2015) 29(1):35–44. 10.1016/j.tmrv.2014.09.003 25447555

[45] Darshana T Rees D Premawardhena A. Hydroxyurea and blood transfusion therapy for sickle cell disease in south Asia: inconsistent treatment of a neglected disease. Orphanet J Rare Dis. (2021) 16(1):148. 33757549 10.1186/s13023-021-01781-w PMC7989007

